# Multiplexed fluidic circuit board for controlled perfusion of 3D blood vessels-on-a-chip†

**DOI:** 10.1039/d2lc00686c

**Published:** 2022-12-09

**Authors:** Mees N. S. de Graaf, Aisen Vivas, Dhanesh G. Kasi, Francijna E. van den Hil, Albert van den Berg, Andries D. van der Meer, Christine L. Mummery, Valeria V. Orlova

**Affiliations:** Department of Anatomy and Embryology, Leiden University Medical Center 2333 ZA Leiden The Netherlands v.orlova@lumc.nl; Applied Stem Cell Technologies, University of Twente 7500AE Enschede The Netherlands; BIOS Lab on a Chip Group, MESA+ Institute for Nanotechnology, Technical Medical Centre, Max Planck Institute for Complex Fluid Dynamics, University of Twente 7500AE Enschede The Netherlands; Department of Human Genetics, Leiden University Medical Center 2333 ZA Leiden The Netherlands; Department of Neurology, Leiden University Medical Center 2333 ZA Leiden The Netherlands

## Abstract

Three-dimensional (3D) blood vessels-on-a-chip (VoC) models integrate the biological complexity of vessel walls with dynamic microenvironmental cues, such as wall shear stress (WSS) and circumferential strain (CS). However, these parameters are difficult to control and are often poorly reproducible due to the high intrinsic diameter variation of individual 3D-VoCs. As a result, the throughput of current 3D systems is one-channel-at-a-time. Here, we developed a fluidic circuit board (FCB) for simultaneous perfusion of up to twelve 3D-VoCs using a single set of control parameters. By designing the internal hydraulic resistances in the FCB appropriately, it was possible to provide a pre-set WSS to all connected 3D-VoCs, despite significant variation in lumen diameters. Using this FCB, we found that variation of CS or WSS induce morphological changes to human induced pluripotent stem cell (hiPSC)-derived endothelial cells (ECs) and conclude that control of these parameters using a FCB is necessary to study 3D-VOCs.

## Abstract 2

We developed a fluidic circuit board for simultaneous perfusion of up to twelve 3D vessels-on-a-chip under comparable wall shear stress using a single set of control parameters despite high intrinsic sample diameter variation.

## Graphical abstract

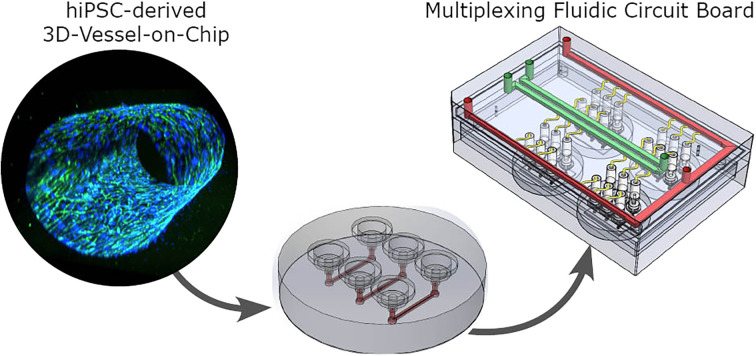

## Introduction

Blood vessels are crucial for distributing vital nutrients and removing metabolic waste products from the human body and are crucial for proper organ function.^1,2^ Diseased vasculature can lead to a wide variety of diseases including arteriosclerosis,^3^ aneurysms^4^ and sepsis.^5^ One way to recapitulate the functionality of vasculature accurately, is to reconstruct the complex microenvironment of blood vessels *in vitro* using microfluidic technology to generate ‘vessel-on-chip’ (VoC) models.^6,7^

Blood vessels are lined with endothelial cells (ECs) surrounded by pericytes or smooth muscle cells that are collectively called mural cells. Mural-EC interaction is vital for blood vessel function and is an important factor in the onset of complex multicellular diseases.^8,9^ Furthermore, mural cells mechanically support ECs and enable efficient distribution of blood flow by contraction or dilation of the vessel. Inside organs, ECs form a tissue-specific-barrier and control the nutrient flux from the blood to tissue.^10^ Additionally, ECs are important in mediating the inflammatory response *via* regulating leukocyte trafficking.^11,12^

The EC phenotype is highly plastic and is continuously influenced by many biological cues that include the surrounding tissue cells, extracellular matrix (ECM), and drugs.^13,14^ In addition, haemodynamic forces, like the wall shear stress (WSS) and circumferential strain (CS), are important modulators of EC phenotype and function.^15,16^ Haemodynamic forces vary across blood vessels of different diameters and depend on the location of the vessel in the vascular “tree”.

WSS is the force parallel to the blood flow, exerted on the vessel wall due to viscous forces. WSS depends on the flow rate, luminal diameter and the viscosity of blood.^17^ In healthy blood vessels, ECs are exposed to unidirectional laminar flow with mean WSS ranging from 0.1 Pa to 5 Pa *in vivo*, depending on the blood vessel location in the vascular bed, while, in larger arteries flow can also be turbulent because of increased diameters or bifurcations. ECs have multiple molecular mechanisms that can sense and react to changes in WSS by modulating the cellular response.^18^

CS is the deformation of the blood vessels due to pressure differences between the lumen and surrounding tissue and results in the stretching of ECs.^19^ Due to the cardiac cycle and location in the vascular tree, CS can be either cyclic (aorta and arteriole) or constant (capillaries).^20^ CS is defined as Δ*L*/*L* where *L* is the perimeter length of the vessel wall. CS is known to modulate many endothelial processes like, actin reorganization and focal adhesions,^21^ matrix remodelling^22^ and apoptosis.^23,24^ Typical physiological values can be up to 15% and higher values are considered pathological.^25^

Microfluidic technology allows engineering of three-dimensional (3D) blood-vessels-on-a-chip (VoC) that combine not only various cell types, but also ECM and haemodynamic forces. Different methods to engineer 3D-VoCs have been developed for various purposes and include, but are not limited to, 3D-bioprinting,^26,27^ template casting^28,29^ and cellular self-assembly.^30,31^

The top-down engineering of 3D-VoCs is preferred over self-assembly methods when controlled haemodynamic forces are required. The resulting 3D-VoCs are versatile and can support a range of different ECM and cell types and can be used to investigate the endothelial barrier function,^29^ tissue-specific and mural-cell interaction^32,33^ and leukocyte migration.^34^

To introduce haemodynamic forces in VoCs, controlled flow is applied using microfluidic pumps.^29,35,36^ The programmed flow rate depends on the luminal diameters and is set to match the *in vivo* situation of interest. Using controlled microfluidic pumps, WSS of the capillary, for instance, can be mimicked in a larger, more tractable 3D model.

Nevertheless, increasing the throughput of perfusion systems remains a challenge. To do this, methods are being developed to increase the throughput of perfusion systems by multiplexing fluidic circuits. The fluidic circuit board (FCB) offers a practical solution and various FCBs are being developed to multiplex OoCs. FCBs are microfluidic analogues of printed circuit boards (PCBs) and are designed to simplify the perfusion of organ-on-chip devices by combining multiple microfluidic components on a microplate footprint.^37^ We previously described different FCBs to multiplex perfusion in OoCs from highly complex controlled circuits, to simple fluidic “distributing” circuits.^38–40^ Haemodynamic parameters in 2D-OoCs can be controlled precisely when the dimensions of the culture chambers are known. Therefore, in 2D-OoCs, a single set of perfusion parameters is sufficient for the accurate flow control. On the other hand, controlling hemodynamic parameters is challenging in 3D-OoCs when the exact dimensions are not known due to a high intrinsic variability.

Template removal,^20^ cell-seeding,^33,36^ hydrogel structure,^41^ incubation times^28^ and biological response^34^ can contribute to diameter variance which results in complexity of the multiplexed perfusion. The compliance of the patterned ECM further increases diameter variation when perfused, as fluidic pressure will also deform soft materials from which the channels are made.^36^ To account for these significant luminal diameter differences, individual control parameters need to be corrected to ensure equal WSS among the different replicates to maintain equal experimental conditions.

In this study, we demonstrated a simple solution to circumvent intrinsic diameter variation of 3D-VoCs to induce equal haemodynamics forces using a single set of control parameters. We optimized our previously developed FCB^40^ to perfuse up to twelve 3D-VoCs with variable luminal diameter while maintaining comparable WSS. The FCB described here has several advantages: (1) it maintains stable WSS and CS in 3D-VoCs using a single pressure difference, even if their diameters vary; (2) it allows simultaneous perfusion of up to twelve 3D-VoCs; (3) the FCB allows “plug-and-play” connection of 3D-VoCs and (4) it is fully compatible with standard microscope-stages, allowing automated imaging while being fully functional.

## Experimental

**Fig. 1:**
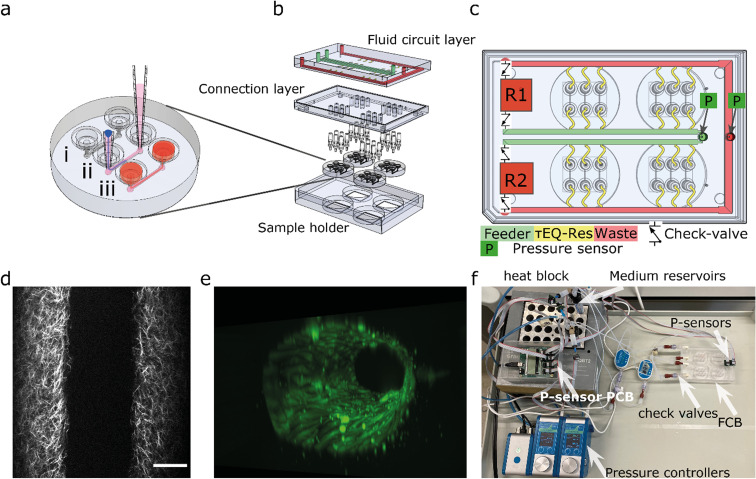
Design of the microfluidic system (a) the round microfluidic devices contain 3 microfluidic channels: (i) microfluidic channels are 1.1 cm long and 500 × 500 μm (wxh); (ii) VFP consists of injection of a viscous collagen mixture (pink) into the channel followed by a droplet of PBS; (iii) pipette-tips are removed and cells can be seeded. (b) Expanded view of the fluidic circuit board, which consists of a fluidic circuit top-layer, a connection layer housing the luer-to-1/16′ barb connectors to four individual devices. (c) The fluidic circuit contains two medium reservoirs (R1,R2) connected to pressure controllers (not shown), a feeder channel (green), *τ*EQ-resistors (yellow) and a waste channel (red) check valves are located at the medium reservoirs so recirculation can be achieved by switching high- and low pressure between the reservoirs. (d) 2p-SHG image shows the collagen fibrillar structure that reveals the lumen. (e) 3D reconstruction of TUBA1B-eGFP-ECs used for this study. (f) Photograph of the tested fluidic circuit board, connected to the external medium reservoirs in a heat block. Scalebar 100 μm.

To multiplex the perfusion of the 3D-VoC devices, we designed a FCB where multiple VoCs can be connected simultaneously in a parallel fluidic circuit (Fig. 1). This resolves the complexity commonly experienced when connecting multiple VoCs to a microfluidic set-up and, in addition, reduces the tedious task of cutting individual pieces of tubing with varying lengths to connect the different system components.

The fluidic circuit consists of a main feeder channel (Fig. 1a, FCB green channel) that has individual branches towards the VoCs (yellow channels). The fluidic flow is then collected in a central waste channel (red channel) that directs the flow towards the opposite medium reservoir. In the central feeder and waste channels, two pressure sensors are inserted that act as the process variable for a proportional-integral-derivative (PID) controller run by custom software.^35^ The FCB used can connect up to four VoC devices, each containing three microfluidic channels (Fig. 1b).

To circumvent the 3D-VoC diameter variation, samples are connected in parallel from a central feeder channel *via* branching channels that have specific dimensions, referred to as equilibrating shear-, or *τ*EQ-resistors (Fig. 1c, yellow). These *τ*EQ-resistors are fluidic resistors that are optimized to ensure that WSS is stable for a specific range of diameters of 3D-VoCs using eqn (S8).† The design principle is flexible, as it can be optimized for all different diameter ranges. Their functionality can be explained using an electrical circuit analogy.^42^ When a constant Δ*P* is applied between the feeder and waste channel, two hydraulic resistances (*R*_h_) are present in each individual VoC channel: (1) the 3D-VoCs and (2) the respective τEQ-resistor. The flow rate can then be predicted using eqn (S2†) where the flow rate is determined by the sum of both *R*_h_. When a 3D-VoC has a diameter in the lower range of the expected variation, the summed *R*_h_ will be high, leading to a lower flow rate. When the diameter is in the higher range, the *R*_h_ of the 3D-VoCs is lower and therefore the summed *R*_h_ will be lower, thereby increasing the flow rate through this VoC. The result is that, at a constant Δ*P*, 3D-VoCs with a small diameter will receive less fluid flow than 3D-VoCs with a large diameters, thereby maintaining the WSS within a narrow window of variation for all 3D-VoCs.

The required Δ*P* for a desired WSS can be calculated using the following equation assuming uniform lumen diameters:1
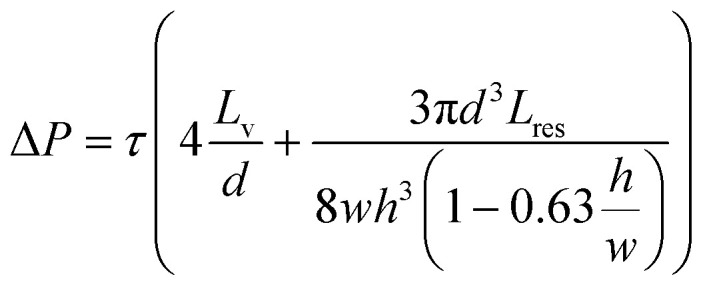
where Δ*P* is the required pressure setting [Pa], *τ* is the intended WSS [Pa], *L*_v_ the 3D-VoC length [m], d the 3D-VoC diameter [m], *L*_res_ length of the *τ*EQ-resistor [m], *w* width and *h* height of the *τ*EQ-resistor [m]. The CS is controlled by controlling the back pressure of the receiving fluidic reservoir until the *P*_FCB_ achieves the desired internal pressure, while maintaining the same Δ*P*.

The microfluidic circuit is designed to recirculate cell culture medium using a passively controlled fluidic circuit analogue to a Graetz-bridge (Fig. 1c). A more detailed description of the functionality is provided elsewhere.^40^ In short, unidirectional flow is achieved by placing 4 microfluidic check valves in a fluidic circuit similar to that of a Graetz-rectifier bridge. When the pressure commands are inversed as the reservoir empties, flow remains unidirectional at the samples without the use of actively synchronized electronic valves.

## Materials and methods

### Fabrication of the fluidic circuit board

The FCB was designed in SolidWorks® and composed of two cast 10 mm and one 15 mm polymethylmethacrylate (PMMA) plates (Altuglass). All connecting channels and fittings for Luer-slip connectors were milled with a CNC micro mill (Datron Neo, Datron AG). After milling, The FCB was assembled as previously described.^39^ Briefly, both layers of the FCB were thoroughly cleaned using industrial cleaning wipes (Adolf Würth GmbH & Co), deionized water, absolute ethanol (Sigma-Aldrich), and propanol (Sigma-Aldrich). A solution of acetone (Sigma-Aldrich) in absolute ethanol at a volume ratio of 1 : 10 was added on top of the connection layer slab and the complementary channel layer slab was then pressed onto the connection layer slab and aligned using pins (DIN 7 – ISO 2338). The assembled FCB was subsequently pressed at 1 kN at 55 °C using a hydraulic press (model 3889, Carver Inc.). Connection between the microfluidic devices and FCB was achieved by inserting barb connectors (male luer-to-barb 1/16′′, IDEX) at the appropriate location (Fig. 1b).

The FCB was tested with off-board medium reservoirs, check-valves and flow sensors to reduce fabrication steps (Fig. 1f). A full list of components required is shown in Table 1. Two 15 mL falcon tubes were used as reservoirs, using 4-port pressure caps (Elveflow). Four check-valves with low cracking pressure (12 mbar, Masterflex) were connected to the FCB. Low-resistance polytetrafluoroethylene-tubing (PTFE, ID 800 μm) was used to connect all external fluidic components. For long-term cell culture, an alternative circuit is used with the same principle using a single flow sensor (Fig. S1†).

List of components

**Table:** 

Electronic component	Qty.	Manufacturer	Product SKU:
Flow EZ-line up 345 mbar	2	Fluigent	LU-FEZ-345
Link-up module	1	Fluigent	LU-LNK-0002
Flow sensor L	1 or 2	Fluigent	FLU-L-D-FDG
Pressure sensors	4 or 6	Honeywell	MPRLS0300YG0001B
Pressure sensor printed circuit board (PCB)	1	Custom	
MCU	1	Custom

**Table:** 

Optional:			
Pressure source 1.2 bar	1	Fluigent	FLPG005

**Table:** 

Fluidic component			
Microfluidic reservoir for 15 mL falcon tube-S (4 port)	2	ELVEFLOW	LVF-KPT-S-4
15 ml reservoirs	2	Greiner-Bio one	
Check valves	4	Master flex	MF-30505-92
Luer-male to 1/16 barb	24	IDEX	CIL-P-854
¼-28-Female to male Luer adapter	4	IDEX	CIL-P-655-01
¼-28-Female to female Luer lock adapter	4	IDEX	CIL-P-678
PTFE tubing – 1/16′′ OD X 1/32′′ ID*		ELVEFLOW	LVF-KTU-15
Female luer bulkhead 1/4–28 thread, to 1/16′′ hose barb	2	Cole-Parmer	45 508–30

**Table:** 

Optional:			
3-Port valve	2	Masterflex	HV-30600-41
*Y*-Connectors	2	IDEX	CIL-P-512

Gauge pressure sensors (Honeywell, MPR-0300YG) were used to measure the pressure difference at the FCB and the reservoir liquid level. Sensors were directly inserted on the FCB at the designated locations (Fig. 1c). Pressure sensors were inserted remotely in the Falcon tubes using a piece of tubing. All pressure sensors were connected using ribbon wires to a micro control unit and connected to the controlling computer using USB. A custom python-based PID-controller was developed to control two 345 mbar LineUp EZ-flow (Fluigent) pneumatic pressure controllers.^35^

### 3D computer fluidic dynamics (CFD) simulation

The WSS characterization was run in SolidWorks flow simulation module (Dassault Systèmes, France), using a viscosity of 0.79 [mPa s], with no gravity and with no slip condition. For this simulation we assumed no interstitial flow. Two simulations were run to characterize the WSS and pressure distribution. First, a simulation including the entire domain of the FCB was carried out. From this simulation, the relative pressure at the inlets of the 3D-VoCs were determined. These pressures were used as input for the second simulation where only the lumens of the 3D-VoC were considered. This strategy enabled an increased mesh density for the simulation and determination of an expected WSS among the different lumen diameters considered.

### Fabrication of microfluidic devices

Microfluidic devices were fabricated from polydimethylsiloxane (PDMS, Sylgard 184, Dow Corning) using injection moulding, as opposed to conventional soft lithography methods.^43,44^ Injection moulding allows the imprinting of media reservoirs, which act as leak-tight connections to the FCB using standardized barb connectors (Fig. S2a†). The microfluidic devices have the same dimensions as standard 35 mm Petri dishes, allowing easy handling and connecting to the FCB. Injection-moulds for the microfluidic devices were designed in SolidWorks and fabricated in PMMA using micro milling. Dimensions of flow channels were as previously described^33^ (1.1 cm × 500 μm × 500 μm; *l* × *w* × *h*, Fig. S2a†). The procedure is shown in Fig. S2b.†

PDMS and base agent were mixed 10 : 1 (w : w) with curing agent and degassed at room temperature. The degassed PDMS was transferred to a syringe. Prepared syringes were kept at −20 °C until use for up to 3 months. The PMDS was allowed to warm to room temperature before injection. The injection mould was assembled using six neodymium block magnets (N42, 1.3 T, approximately 60 N per magnet, Webcraft GmbH) and the PDMS was slowly injected (Fig. S2b (2)†). The filled injection-mould was set vertically at room temperature (19–22 °C) overnight for the initial crosslinking to minimize shrinkage. Afterwards, the PDMS was further cured at 75 °C for 60 minutes. The PDMS was carefully peeled off and excess PDMS was cut off. Post-production examination of microfluidic devices showed a small number of devices with inlet defects and these devices were subsequently discarded or removed from analyses (Fig. S2c†).

The assembly of the microfluidic devices was carried out as previously described with modifications.^33^ PDMS devices and round cover glasses (#1.5, ∅ 30 mm Thermo scientific) were surface-activated using air plasma (45 s, 50 Watt at 60 Pa, CUTE-Femto Science) and contact bonded using light pressure. Immediately after contact bonding, microfluidic channels were functionalized to covalently bind collagen I. First, a 0.1% (v/v) 3-aminopropyl-triethoxysilane (APTES, Sigma-Aldrich), 0.005% distilled water (Gibco®) was prepared in methanol (Technical grade, Sigma-Aldrich), injected in the channels and incubated for 30 minutes at RT.^45^ Channels were thoroughly rinsed with methanol and dried using a nitrogen-gas flow. Subsequently, the devices were incubated at 110 °C for 30 min on a hotplate. Next, channels were injected with 5% (v/v) glutaraldehyde (Sigma-Aldrich, in distilled water) and incubated at RT for 30 minutes. Channels were thoroughly rinsed with distilled water, dried under nitrogen gas flow and baked at 75 °C for at least 2 hours.

### Lumen patterning using viscous finger patterning

Collagen scaffolds were generated as previously described with a minor modification.^33^ Briefly, the 7 mm tip ends of P10 pipette tips (Greiner Bio-One #741015) were used as a driving tip and intact tips as “receiving” tips for patterning (Fig. 1aii). The collagen I hydrogel was prepared as follows: (1) reconstitution buffer was prepared using M199 medium 10× (Gibco®), 4-(2-hydroxyethyl)-1-piperazineethanesulfonic acid -buffer (HEPES, ThermoFisher, final concentration 10%), sodium bicarbonate (ThermoFisher, final concentration 2.2 g L^−1^), distilled water and 1 M sodium hydroxide according to the manufacturer's instructions; (2) collagen I high concentration (Corning, cat# 354249, 11.0 mg ml^−1^) was aliquoted and the quantity was verified using weight; (3) the reconstitution buffer was added to the collagen I stock to achieve the final concentration of 5 mg ml^−1^; the solution was thoroughly mixed, centrifuged to remove air bubbles and transferred to a 1 ml syringe (BD Luer-Lok™). The collagen I mixture was sequentially injected *via* the receiving tip using a plastic blunt needle (Techcon, 20G) until the meniscus of the collagen I mixture reached the outlet of the driving tip. Subsequently, 3.5 μl of PBS was pipetted on top of the collagen in driving tip to initiate patterning using a multi-dispensing pipet. Immediately after lumen patterning, microfluidic devices were incubated for 30 minutes at 37 °C in a humidified incubator. After collagen gelation, endothelial growth medium-2 (EGM-2, Promocell) supplemented with penicillin–streptomycin (PenStrep, Thermofisher, final concentration 25 units per ml), was pipetted in the receiving tip and devices were further incubated overnight at 37 °C. Prior to cell seeding, the driving and receiving tips were removed in a smooth twisting motion.

### Differentiation and expansion of hiPSC-ECs

Alpha-tubulin- monomeric enhanced green fluorescent protein-hiPSCs (TUBA1B-mEGFP, cell line ID: AICS-0012 cl.105, https://hpscreg.eu/cell-line/UCSFi001-A-2) were obtained from the Allen institute.^46^ hiPSC-ECs were differentiated as previously described.^47^ Briefly, TUBA1B-mEGFP-hiPSCs were maintained in TeSR™-E8™ medium on vitronectin-coated 6-well plates and seeded at day (−1). Twenty-four hours after seeding, E8 medium was replaced with B(P)EL medium supplemented with 8 μM CHIR. On day 3, the medium was replaced with B(P)EL medium supplemented with 50 ng ml^−1^ VEGF (R&D systems) and 10 μM SB431542 (Tocris Bioscience); cells were refreshed with the same medium on days 7 and 9. hiPSC-ECs were isolated on day 10 using CD31-Dynabeads™ (Invitrogen), expanded for 3 days and cryopreserved. hiPSC-ECs from cryo-preserved batches were used in all experiments. They were thawed and expanded in endothelial cell-serum free medium (EC-SFM, Gibco, cat. No. 11111-044) supplemented with 1% human platelet poor serum, FGF2 (20 ng mL^−1^) and VEGF (30 ng mL^−1^), on a 0.1% gelatine-coated T-75 culture flask. hiPSC-ECs used in 3D cell cultures were at passage 2.

### Seeding of collagen scaffolds with hiPSC-ECs

HiPSC-ECs were harvested using TrypLE™ (Thermo Fisher), and resuspended at a concentration of 15 × 10^6^ cells per ml in EGM-2 supplemented with 50 ng mL^−1^ VEGF and PenStrep. 5 μl of cell suspension was injected using a multi-dispensing pipette with a maximum flow rate of 100 μl min^−1^. After injection, microfluidic devices were placed on a slow rotator (1 RPM, channel longitudinal axis in line with rotating axis) and rotated for 1 to 2 hours at 37 °C until all cells were attached and completely covered the collagen scaffold. Medium was refreshed and samples were placed inside a humidifier-box to prevent medium evaporation and incubated at 37 °C, 5% CO_2_. Medium was refreshed every 24 hours with EGM-2.

### Measurement of *τ*EQ-functionality

Samples were prepared as described above and individually connected to the fluidic pump using a total 52 cm of microfluidic tubing divided over two tubes (Fig. S4,† ID 800 μm). The pressure difference was controlled using the custom PID-software^40^ and the flow rate measured using a flow rate sensor (Fluigent, size L). Flow rate was allowed to stabilize and a 5 second average was used as the measured value. The 3D-VoC diameter was determined using widefield microscope and the WSS was calculated using eqn (S3†) and compared to the theoretical value (eqn (1)).

### Particle image velocity (PIV)

Cell culture medium was supplemented with 25 × 10^6^ fluorescent beads per ml (Thermo Fisher, 1 μm). Bead displacement was captured using a Leica DMI6000 equipped with a Zyla 4.2 sCMOS and a 10× objective. Maximum framerate of 286 frames per second was achieved using binning of 3 × 3. PIV was performed using a custom script using the OPENPIV library.^48^ The following settings were used to analyse all frames: binary threshold: 100; interrogation window: 24 px; search window: 48 px; overlap: 12 px. All vector fields were combined and filtered based on median of the location to improve accuracy. Using the maximum velocity in the centre of the lumen the average velocity was calculated assuming Poiseuille flow to calculate the flowrate with eqn (S3).†

### Assessment of 3D scaffold and lumen expansion

Unseeded collagen scaffolds were individually connected to the fluidic perfusion system and placed upside down on an upright ZEIS LSM710 NLO microscope equipped with a multi-photon laser tuned at 810 nm. Two-photon second harmonic generation (2P-SHG) images were acquired using a non-descanned detector at 380–430 nm. Internal pressure was controlled using the pressure pumps with no flow (*i.e.* no pressure difference). Pressure was varied between 0 mbar and 345 mbar. At every pressure step, a 3D stack of images of the lumen was taken.

Scaffolds were seeded with TUBA1B-mEGFP-ECs, and imaged using a Leica DMi8 microscope equipped with a Dragonfly® spinning disk (pinhole:40 μm) (Andor). A HC PL APO 20x/0.75 IMM CORR CS2 objective was used with water as immersion medium. Pressure was varied between 0 mbar and 345 mbar. At every pressure step a 3D stack of images of the lumen was taken.

### Measurement of collagen I scaffold compliance

Diameter expansion was determined in pixels at the widest slice of the 3D-2p-SHG stack using VasoTracker software using the average of 50 lines across the field of view.^49^ The strain was calculated using eqn (2):2
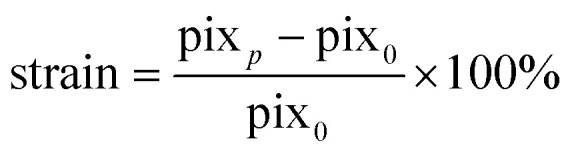
Where pix_*p*_: measured pixel length at pressure *P*, pix_0_ pixel length at *P* = 0.

### Measurement of 3D-VoC compliance

3D-VoCs were connected to the FCB and placed on an EVOS widefield microscope. The GFP signal of the TUBA1B-mEGFP was captured at every pressure step with the centre of the lumen in focus. Diameter expansion was determined in pixels using VasoTracker software using the average of 50 lines across the field of view. Vascular compliance was calculated using eqn (2).

### Live-cell imaging of 3D-VoCs

Prior to imaging, 10 μl of EGM-2 with anti-VE-cadherin (CD144-mouse anti-Human Alexa-647, BD Bioscience, diluted 1:200) and Hoechst—33 342 (Thermo Scientific, 1 μg ml^−1^) were injected into the lumen of 3D-VoCs and incubated for 30 min at 37 °C. Next, 3D-VoCs were connected to the FCB and flushed with fresh medium. Samples were imaged using Leica DMi8 microscope equipped with a Dragonfly® spinning disk (pinhole:40 μm) (Andor). A HC PL APO 20×/0.75 IMM CORR CS2 objective was used with water as immersion medium. This objective was combined with 2× camera magnification to enhance lateral resolution. An iXon CCD camera was used to record the signal.

### Long-term recirculation

3D-VoCs were prepared as described above and cultured under static conditions for 48 hours. The FCB was primed with EGM-2 + PenStrep and the 3D-VoCs were connected. The air compressor was placed inside of a dry incubator (5% CO_2_) and the 3D-VoCs were perfused using a pressure difference of 1 mbar and 50 mbar of *P*_FCB_ for 24 hours using the custom PID-software.^40^

### Immunofluorescence staining of 3D-VoCs

4% paraformaldehyde (PFA) solution was injected into the lumen of 3D-VoCs and incubated for 15 minutes at RT. Cells were subsequently permeabilised using 0.5% (v/v) Triton-X 100 in PBS (without Ca^2+^ and Mg^2+^) for 10 minutes at RT. Samples were blocked with 2% BSA in PBS (without Ca^2+^ and Mg^2+^) (w/v) for 30 minutes at RT. Alexa Fluor™ 647 phalloidin (Thermo fisher, final concentration 165 nM) was diluted in 1% BSA in PBS and 10 μl was injected into the channels. Channels were incubated at RT for 1 hour and washed three times with PBS (without Ca^2+^ and Mg^2+^) for 10 minutes. Cell nuclei were stained using 4′,6-diamidino-2-phenylindole (DAPI, 300 nM, Thermo fisher) for 10 minutes at RT. Samples were stored in the dark at 4 °C until imaging using a Dragonfly® spinning disk (pinhole size:40 μm, Andor).

### Quantification of cell junctions

The tortuosity index was calculated to quantify cell junctions.^50,51^ First, the VE-cadherin images acquired at low pressure (0 mbar) and high pressure (345 mbar) were set at threshold and “skeletonized’ to visualize cell–cell junctions. The resulting skeletonized imaged were analyzed using the AnalyzeSkeleton plugin.^51^ The length of cell borders (Lb) divided by the branch length (Euclidean distance) (Le) gives the tortuosity index. Cell border lengths greater than 10 μm were included in analysis.

### Statistics

Statistical analysis was performed using Microsoft office Excel Analysis-ToolPak.

For the scaffold compliance analysis, a Student's *t*-test assuming unequal variances was used. Results are presented as mean ± 99% confidence interval.

For the tortuosity index analysis, a Student's *t*-test assuming equal variances was used. Results are presented as mean ± standard deviation.

A coefficient of variance (CV) was calculated with the following equation: standard deviation/mean to quantify the variation.

## Results and discussion

### Design of the fluidic circuit board (FCB) for multiplexed and controlled perfusion of 3D vessels on chip (3D-VoCs)

The FCB developed for multiplexing up to twelve 3D-VoCs in parallel is shown in Fig. 1a–c. The fluid circuit consists of two fluidic reservoirs (R1,R2), a feeder-channel (green, inner loop), a waste channel (red, outer loop) and parallel branching channels that distribute the medium towards the individual samples, referred to as *τ*EQ resistors (yellow). The dimensions of these τEQ-resistors can be optimized for any expected sample diameter range using eqn (S9) or (S10†) to minimize WSS for that specific diameter range. The proposed design for this FCB is flexible, with only the length or height of the *τ*EQ-resistors needing adjustment when a different range is required; this can be directly milled in the PMMA. For this prototype, medium reservoirs, check valves and flow sensors were placed off the FCB to reduce the number of fabrication steps, using off-the-shelf components listed in Table 1 and connected using standard microfluidic tubing following the same circuit scheme (Fig. 1f). This fluidic circuit was designed to perfuse four microfluidic chips sharing the same medium. This single fluidic circuit is a simple platform that allows conditioning of as many as twelve 3D-VoCs simultaneously for higher throughput. Furthermore, the fluidic circuit could be adapted for additions in the future, such as combining different tissue-specific 3D-VoCs and other OoCs with shared medium as in the “Body-on-Chip” concept.^52^ Alternatively, microfluidic circuits may be separated by designing a complex pneumatic system,^38^ to be able to apply different chemical conditions on one plate.

Pressure was controlled using two 345 mbar (34.5 kPa, 260 mmHg) pneumatic pressure controllers connected to the medium reservoirs to mimic the full range of the human physiological blood pressure. A custom-written PID-controller was programmed to maintain a constant Δ*P* between feeder and waste channel.^40^ The desired luminal pressure (*P*_lumen_) was controlled by raising the back-pressure of the receiving reservoir. Depending on the tubing and check-valves used, a minimum *P*_lumen_ of 20 mbar is required at 1 Pa of WSS, with a *P*_lumen_ that can range up to 325 mbar. Importantly, the pressure head of the reservoir liquid level influences the minimum pressure up to 8 mbar. Higher WSS up to 5 Pa is possible at the cost of higher minimum *P*_lumen_ and a lower maximum *P*_lumen_.

The microfluidic devices are connected using 1/16′′ barb connectors. To be able to connect the microfluidic devices we used injection moulding to fabricate a direct, leak-tight connection between the devices and the barb-connectors (Fig. S2a†). The injection-moulds used for this work contained some defects at the inlet of some channels resulting in the formation of small PDMS membranes (Fig. S2c†). Complete removal of these defects was not always successful. In some cases, this membrane remained in place after punching, or the inlet was damaged in such way that leakage occurred when pressure was applied. These defects significantly increased the *R*_h_ of the flow path, influencing the WSS. Samples where these defects were present were excluded from flow rate analysis as their dimensions were not fabricated as designed, reducing the total number of samples that could be presented in this study simultaneously.

To determine the range of sample diameters for which the FCB should be optimized, 3D-VoC lumens were patterned with a minimal amount of driving fluid to reach the minimal diameter for the given microfluidic channel. Lumen diameters can be further controlled by varying important parameters like collagen concentration or driving pressure.^32,53^ The scaffold consists of a 5 mg ml^−1^ collagen I hydrogel and was imaged using two-photon-second harmonic generation (2P-SHG) (Fig. 1d). The diameter of collagen scaffolds was measured 202 μm ± 10 μm (mean ± standard deviation, *n* = 13) (Fig. S3†) which was expected based on the geometries of the microfluidic channels used (500 × 500 μm) and earlier results.^32,33^ The diameter ranged between 185–220 μm with an outlier of 244 μm (Fig. S3†). As observed earlier,^33^ lumens expanded during the cell seeding step with the diameter measured 240 μm ± 20 μm (*n* = 16). The origin of this initial expansion is the unintended injection of air-bubbles, which leads to compaction of the soft hydrogel scaffolds. The luminal diameters of seeded scaffolds ranged from 187 to 290 μm. The diameters of the lumens produced remained constant over the period of 3 days upon static cell culture with medium being refreshed every 24 hours (Fig. S3†). However, depending on the hydrogel scaffolds and cell types used, these luminal diameters might be variable over time.^28,41^ Furthermore, vessel sprouting during the 3 day culture period under the conditions described was rare, at most occasional small sprouts being observed. Using this observed diameter range, we optimized the FCB for lumen diameters in the range of 180 μm to 300 μm.

### Computational modelling of the flow distribution in the FCB

**Fig. 2:**
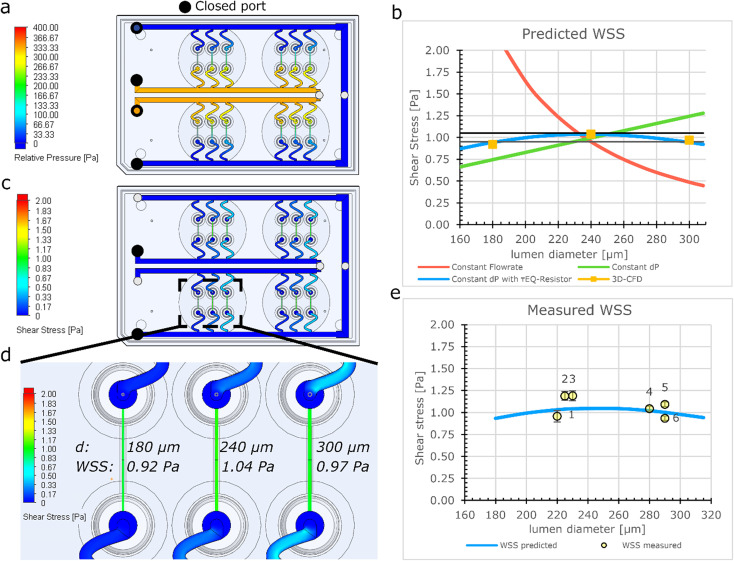
Numerical model of fluidic parameters on a FCB (a) CFD- simulation of the pressure distribution indicates an equal pressure difference over the feeder channel and waste channel along the full length. (b) Modelling WSS using electrical circuit analogy shows high diameter dependence of WSS to flow rate (red plot), linear relationship with fixed pressure (green plot), parabolic relationship when using *τ*EQ-resistance and a fixed pressure (blue plot). This parabolic behaviour narrows the variation over a wide diameter range to 10% difference between the minimum and maximum values compared to a 4-fold or 60% in the other situations. (c) 3D-CFD-simulation of WSS demonstrates that all 3D-VoCs on the fluidic board show similar WSS distribution. (d) Detailed CFD-model of the individual 3D-VoCs having 180 μm (left) 240 μm (middle) and 300 μm (right) shows similar values for WSS across the extremes of diameter. (e) Measured WSS using an optimum *τ*EQ resistor and a ΔP of 3.3 mbar shows a comparable trend as the model value error bars show standard deviation of a 5 second average.

The fluidic circuit of the FCB was designed to: (1) achieve equal pressure difference for all 3D-VoCs and (2) minimize the WSS variation of all samples across a wide range of sample diameters. The fluidic circuit channel dimensions are listed in Table S1.† To achieve an equal pressure distribution across all samples, the feeder and waste channels were designed to have a minimal *R*_h_. To reduce effects of the length difference between feeder and waste loops, channels widths were adjusted to maintain equal *R*_h_. The crude estimated pressure distribution of the 3D-CAD model was validated using the computer fluid dynamics (CFD)- simulation module of SOLIDWORKS®. This showed that an equal Δ*P* can be expected using the designed dimensions across the full length of the channels (Fig. 2a).

To investigate how the WSS of the 3D-VoCs varies within the target diameter range using different *R*_h_ of *τ*EQ-resistors, the diameter dependence of WSS was modelled using electrical circuit analogy (Fig. 2b). A common practice in microfluidics is to use high resistance in branching channels to equalize volumetric flow rates.^54,55^ However, WSS in 3D-VoCs is highly diameter-dependent; when volumetric flow rates are fixed (eqn (S3†)), a 5-fold difference in WSS can be expected between the minimum and the maximum WSS in the previously mentioned, targeted range (Fig. 2b, red plot).

When a constant Δ*P* is applied, with a negligible branch resistance, the flow rate is dictated by the *R*_h_ of the 3D-VoCs. A linear relationship between the WSS and the diameter is expected (eqn (S11†))^56^ which resulted in a 2-fold predicted difference between the minimum and maximum WSS (Fig. 2b, green plot, eqn (S3†)). These plots suggested that there is an optimum *R*_h_ where the WSS of the minimum diameter is equal to the WSS of the maximum diameter with the same Δ*P*. This optimum *R*_h_ of the branching channels (sum of the *R*_h_ of 3D-VoC and *τ*EQ) can be calculated using eqn (S9).† Interestingly, using this optimum *R*_h_ the theoretical WSS-difference between the expected minimum and maximum values was reduced to less than 10% across this large diameter range (Fig. 2b, blue plot, eqn (S12†)). The designed dimensions were modelled using 3D-CFD model to validate the WSS equalizing resistance (*τ*EQ-resistance). The 3D-CFD model included the minimum (180 μm), middle (240 μm) and maximum (300 μm) diameters modelled on all fluidic devices, as these diameters would yield the most extreme WSS-values across the diameter range (Fig. 2c and d, Video S1†). The 3D-CFD simulation corresponded with eqn (S12†) (Fig. 2b, yellow squares) demonstrating that designed dimensions function as a *τ*EQ-resistor.

Next, this *τ*EQ-resistance functionality was tested to verify a measurable effect. Microfluidic tubing was cut and placed between the pressure sensors and the sample (Fig. S4†). A total length of 52 cm was determined using eqn (S10†) to match the *R*_h_ of the designed *τ*EQ-resistance. Prior to cell seeding, half of the patterned scaffolds were intentionally widened by injecting air bubbles to generate lumens with smaller- (220–240 μm) and larger (280–300 μm) diameters. Samples were individually connected and 3.3 mbar Δ*P* was applied between the sample and the resistor tubing using the custom software. Flow rate was directly measured using a flow rate sensor, the WSS was calculated using eqn (S3†) and compared with the computational model (Fig. 2e). An average WSS of 1 Pa was found (*N* = 6), which was as predicted with the eqn (S11†) (Fig. 2c, orange plot). A coefficient of variance (CV) of 9.4% in WSS was observed across the measured 3D-VoCs. Differences between the model and experimental data may be due to interstitial flow or small differences in vertical length of the measured lumen as shown in Fig. S2a.† A discrepancy between set- and measured flow rates has been observed earlier;^33^ however, it was not significantly larger than the accuracy of the set flow rate and therefore we concluded that the interstitial flow cannot explain this difference entirely. The length variation stems from the manual insertion of the pipette tips which can introduce luminal length variation of approximately 1–2 mm (Fig. 1aii). 2 mm difference in length may result in up to 12% difference in WSS which explains the observed discrepancy. Improving the microfluidic chip design to increase the accuracy of the pipette tip placement could help in reducing this luminal length variation.

### Experimental validation of the flow distribution on the FCB

**Fig. 3:**
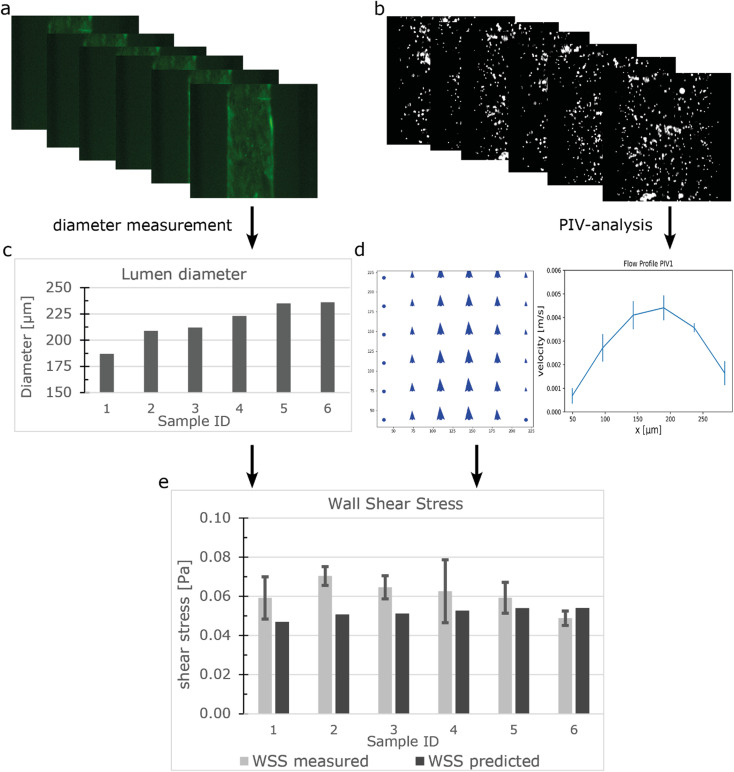
Measuring the flow distribution and WSS using μPIV (a) GFP signal from hiPSC-ECs in the lumens was captured using widefield microscopy. (b) Fluorescent beads were perfused and beads displacement was captured with 286 fps. (c) Luminal diameter was measured. (d) Vector fields were constructed using the beads displacements and the maximum velocity was determined at the Centre of the lumen. (e) WSS was calculated using the diameter and velocity profile. Error bars show 99% confidence interval of a vector field.

Particle image velocity (PIV) was performed to determine *in situ* the flow distribution of the individual samples connected to the FCB using the workflow shown in Fig. 3. The luminal diameters were measured using the GFP signal from the beads and ranged from 187 μm to 236 μm (Fig. 3a and c). Perfusion was performed using a Δ*P* of 0.15 mbar (expected WSS ± 0.052 Pa). 100 frames were captured at 286 frames per second and analysed using a custom Python script using the OPENPIV library (Fig. 3b, S5†). Combined with the diameter, the local WSS was calculated using equation eqn (S3†) with viscosity of medium containing 2% serum at 37 °C being 0.79 [mPa s].^57^ The measured WSS of individual samples had an average of 0.061 Pa and a CV of 12% (*n* = 6), which was slightly higher than the predicted value (Fig. 3e). However, this could be attributed to the previously mentioned length variation between samples.

A limitation of the presented FCB is the inability to monitor the flow rate of individual 3D-VoCs in real time. PIV is not a practical method for normal experimental settings, as it would require the continuous imaging of 12 samples simultaneously to measure flow rate using highly specialized microscopic settings. By integrating multiple CMOS-chip based flow rate sensors in the branching channels, like the Sensirion LPG10 (Sensirion GmHb), individual flow rates can be logged in real time to improve the experimental quality control throughout long-term perfusion experiments at the expense of increasing the complexity of the system.

### Assessment of endothelial cell responses to haemodynamic forces in 3D

To demonstrate the utility of the FCB for biological research we used the FCB to investigate the response of 3D-VoCs to haemodynamic forces in parallel. We first investigated EC-responses to CS induced by application of the intraluminal pressure without flow. Collagen scaffolds without cells were used as a control. First, the expansion of the lumen was investigated in 3D. Collagen scaffolds were imaged with an up-right intravital microscope using 2P-SHG. 3D-VoCs seeded with TUBA1B-mEGFP-hiPSC-ECs cells were imaged using a spinning disk confocal microscope. A gradual increase in the luminal pressure, without application of flow (*i.e.* same pressure at the inlet and outlet), was used.

**Fig. 4:**
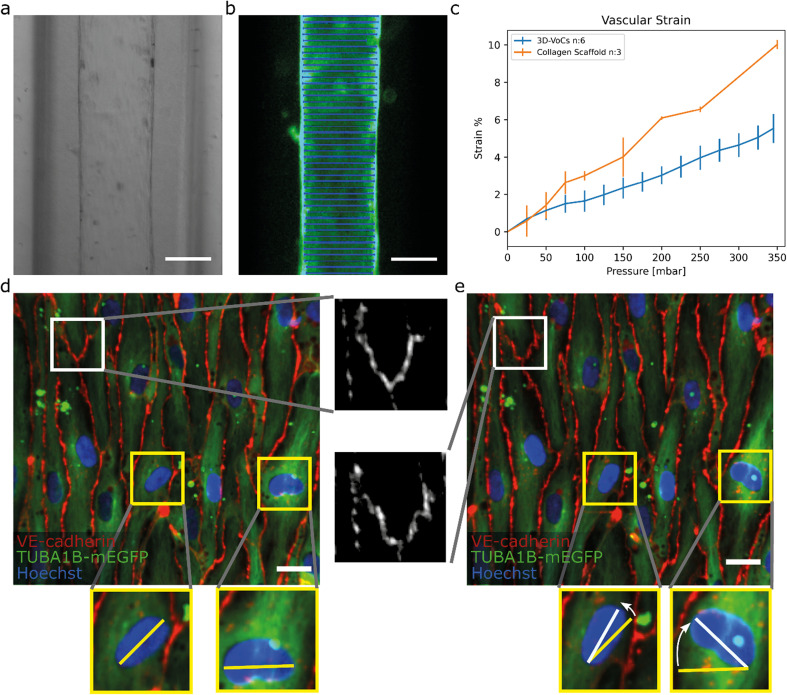
TUBA1B-hiPSC-ECs under circumferential strain (a) brightfield image of a 3D-VoCs in a 500 μm wide fluidic channel. (b) GFP-fluorescent signal of a lumen measured using the VasoTracker software (blue lines). (c) Strain curve of the 2p-SHG scaffold only and seeded scaffolds using the widefield fluorescent signal shows a significant effect of the endothelial monolayer, bars are 99% ci. (d) Confocal reconstruction of live TUBA1B-eGFP-ECs (green) co-stained for adherens junctional marker (VE-cadherin, in red) and nuclei (Hoechst, in blue) at pressure = 0 mbar, white zoomed panel show continuous adherens junctions and yellow zoomed-panels show non-aligned nuclei. (e) Confocal reconstruction of the same region at pressure = 345 mbar shows rapid alignment of unaligned cell nuclei, adherens junctions show the formation of a zig-zag pattern, implying overstretching of the cellular monolayer without rupturing. Scalebar: a and b, 150 μm; d and e 20 μm (see also Videos S5–S8† for the animated Videos).

At different luminal pressure points from *P* = 0 to *P* = 345 mbar with 25 mbar increments 3D stacks were imaged (Fig. S6a and S6b respectively, Table S2, Videos S2 and S3†). Manual diameter measurements showed that both the bare scaffolds and seeded scaffolds expanded symmetrically and therefore the total strain caused by the luminal pressure change could be analysed by measuring the diameter-change using a widefield microscope (Fig. 4a and b).

For this, 3D-VoCs were simultaneously connected to the FCB and imaged using a widefield fluorescent microscope at different luminal pressure points from *P* = 0 to *P* = 345 mbar with 25 mbar increments. For the collagen scaffolds the middle slice of a 2p-SHG 3D stack was used, as the widefield image of collagen did not have enough contrast to determine the collagen border accurately (Fig S6c†). VasoTracker software was used to generate a strain curve of lumens without cells (collagen scaffold, *n* = 3) and with cells (3D-VoCs, *n* = 6) (Fig. 4c). The strain curve shows that the presence of the EC-monolayer significantly reduced the observed strain compared to the scaffolds without cells. At a pressure of 345 mbar 3D-VoCs exhibited a 5.5% ± 0.8 strain while for scaffold-only conditions, this was 11% ±1.3. It is important to note that walls of the PDMS-channel under both conditions also expand in the measured pressure range, contributing to the total measured compliance (Fig S6c, Videos S4–S6†).

The hiPSC-EC monolayers under CS were investigated in greater detail using spinning-disk confocal microscopy. Cells were cultured for 72 hours under static conditions and live TUBA1B-mGFP-ECs were co-stained for VE-cadherin to visualize the cell junctions and Hoechst to visualize the nuclei. The 3D-VoCs were connected to the FCB and imaged while the pressure of the inlet and outlets was simultaneously increased in a stepwise manner up to 345 mbar, avoiding net fluid flow. Due to photobleaching of the VE-cadherin conjugated fluorophore, it was not possible to image more than 4 frames per region. These sequences showed that VE-cadherin junctions retained their orientation up to 100 mbar of internal pressure (Video S7†). At 150 mbar of luminal pressure (strain of approximately 2%), adherens junctions adopted a zig-zag pattern, indicating overstretching of the EC-monolayer and partial opening of the cell–cell junctions (Fig. 4d and e white inserts, Video S8†). Nevertheless, the hiPSC-EC monolayer remained intact up to the maximum measured internal pressure of 345 mbar. Interestingly, non-aligned cell nuclei quickly aligned with the longitudinal axis of the lumen despite the lack of continuous flow (Fig. 4d and e yellow inserts). The tortuosity index of cell junctions was next calculated by dividing the length of the cell borders (Lb) by the Euclidean distance (Le) length.^50^ We found that the tortuosity index of adherens junctions was significantly higher under pressure of 345 mbar when compared to 0 mbar pressure (tortuosity index: 1.06 ± 0.021 *vs.* 1.09 ± 0.048, *P* < 0.001) (Fig. S7†). When the pressure is released, most junctions return to a more straight morphology, however some dislocated junctions remained present (Fig. S9†). These findings suggested that the EC monolayer cannot stretch to the same extent as *in vivo*.^25^ However, it has been shown that pre-conditioning of cells to strain for longer periods can result in higher compliance of EC-monolayers.^58^ These zig-zag patterns could influence the permeability of the endothelium; however it has been shown that continuous exposure to circumferential strain decreases the permeability.^59,60^ Further detailed investigation on how this circumferential stress influences the endothelial monolayer now be carried out using this FCB.

To investigate the cellular response to WSS, cells were exposed to a Δ*P* of 100 Pa (generating a predicted WSS of 0.3 Pa) with an internal pressure of 50 mbar (approximately 1% of strain) for 24 hours. A well-characterized response of ECs to WSS is the reorganization of the actin cytoskeleton.^61,62^ A venous-like WSS of 0.3 Pa (3 dyne per cm^−2^) has been shown to alter actin morphology of primary venous-ECs significantly.^29,63,64^ By forming actin stress fibres, the ECs can react to haemodynamic forces. We used 3D-CFD simulation to confirm the ability of the FCB design to generate equal WSS for the given samples under the applied conditions (*P*_lumen_:50 mbar, dP: 1 mbar) (Fig. S10a and S10d† blue plot). We also examined the samples under higher pressure to confirm that the resulting WSS is constant regardless of applied pressure (Fig. S10b and S10d,† gray plot). We found that, given the luminal dimension and perfusion parameters measured, the hiPSC-ECs experienced 0.32 Pa with a CV of 4%. If all sample had been perfused using a single flowrate, the CV would have been 23% between samples (eqn (S3†)).

**Fig. 5:**
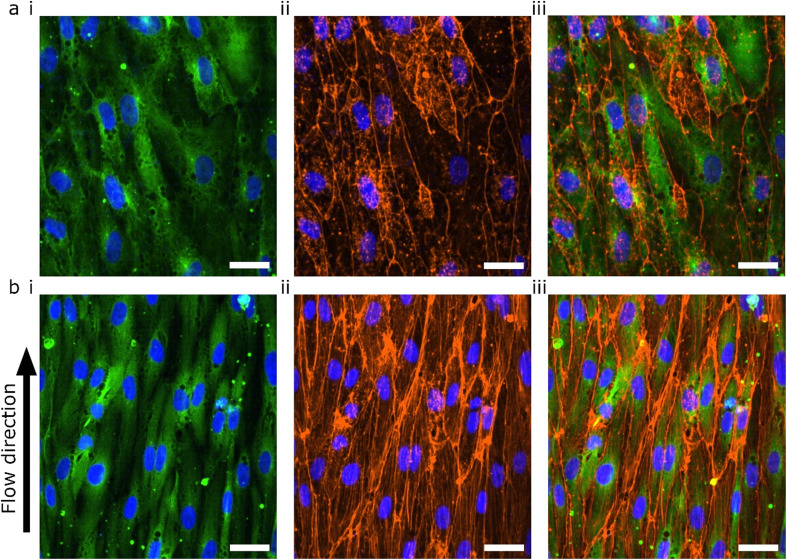
Confocal microscopy of TUBA1B-hiPSC-ECs under WSS (a) hiPSC-ECs cultured in static conditions for 72 hours. (b) hiPSC-ECs cultured in static conditions for 48 hours and 24 hours under 0.3 Pa WSS. Representative images of 7 individual lumens are shown with (i) TUBA1B-eGFP-ECs (green) co-stained for (ii) F-actin (orange) and (iii) merged image. Nuclei are visualized with DAPI (blue) in all images. Scale bars 30 μm.

The lumens were kept for 48 hours in static conditions to promote cell attachment and were then perfused for 24 hours. The 48 hours static- and 24 hours perfusion experiments were compared with 72 hours static. The cells were fixed, permeabilized and stained for F-actin using a phalloidin-conjugated fluorophore (Fig. 5). Both flow and no flow conditions were imaged using the same microscope settings and displayed using the same LUT. In the absence of flow, the F-actin was mainly located at the cortical rim and the overall intensity was low (Fig. 5a). As previously shown, hiPSC-ECs cultured under static conditions in 3D-VoCs showed alignment with the longitudinal axis of the lumen.^33^ On the other hand, 3D-VoCs connected to the FCB without constrictions (*n* = 7) showed both the formation of the actin stress fibres and increased overall intensity, demonstrating that the hiPSC-ECs were able to react to the applied flow much like primary ECs (Fig. 5b).^63,64^ We chose to use F-actin as a proof-of-principle in this study. Additional characterization of morphological changes to WSS, such as investigation of EC polarity would be an alternative to quantify adaptation to WSS in this model.^65^

During perfusion experiments, we often observed delamination of the collagen scaffolds from the PDMS channel walls when using the PDMS surface-coating protocol previously reported.^33^ We hypothesised that this could be due to the sub-optimal coating procedure, as white residue was often observed after coating. We therefore altered the APTES coating procedure by using a low concentration of APTES in methanol.^45^ This optimized protocol resulted in higher bonding of the ECM to the PDMS, so that it could withstand pressures of over 1000 mbar without delamination (Fig S5c, Video S2†).

## Conclusion

Overall, the results presented here demonstrate the ability of the perfusion platform we designed to exert bi-modal mechanical stimulation of up to twelve 3D-VoCs with WSS combined with CS. Due to the fluidic circuit design, the variation in WSS resulting from different 3D-VoCs diameters is minimized, eliminating the need for individual sample control and increasing throughput. To our knowledge, this is the first multiplexed, controlled perfusion system for 3D-VoCs. Combination with a scalable method for generating 3D-VoCs allowed the number of 3D-VoC replicates in a single perfusion experiment to be increased. The FCB we designed is capable of individually controlling WSS and internal pressure using two pressure controllers, while being able to recirculate cell culture medium.

Using this FCB, we demonstrated morphological changes in hiPSC-ECs to both WSS and CS separately, illustrating the need to include and control both stimuli in *in vitro* experiments. Future studies could include a thorough structural and functional analysis the response hiPSC-ECs to both WSS, CS and a combination in physiological and pathophysiological conditions.

We expect that the flexibility of FCBs will help the development of advanced *organ-on-chip* technology with more predictive capabilities.^37–39^ The concept of the FCB presented here allows the number of technical replicates under perfusion to be increased which is important for the development of more complex models. This simple design can be further combined with more advanced concepts to fabricate better and easier to use perfusion platforms.

## Author contributions

Conceptualization: MNSdG, VVO; data curation: MNSdG; formal analysis: MNSdG, AV; funding acquisition: AvdB, ADvdM, CLM, VVO; investigation: MNSdG; methodology: MNSdG, AV, DGK, FEvdH; project administration: VVO; resources: ADvdM, VVO; software: MNSdG; supervision: CLM, VVO; validation: MNSdG; visualization: MNSdG, AV; writing – original draft: MNSdG, VVO; writing – review & editing: all authors.

## Conflicts of interest

The authors declare no conflict of interest.

## Supplementary Material

LC-023-D2LC00686C-s001

LC-023-D2LC00686C-s002

## References

[cit1] Jambusaria A., Hong Z., Zhang L., Srivastava S., Jana A., Toth P. T., Dai Y., Malik A. B., Rehman J. (2020). eLife.

[cit2] Reiterer M., Branco C. M. (2020). FEBS J..

[cit3] Wang K. C., Yeh Y. T., Nguyen P., Limqueco E., Lopez J., Thorossian S., Guan K. L., Li Y. J., Chien S. (2016). Proc. Natl. Acad. Sci. U. S. A..

[cit4] Sheinberg D. L., McCarthy D. J., Elwardany O., Bryant J. P., Luther E., Chen S. H., Thompson J. W., Starke R. M. (2019). Neurosurgical focus.

[cit5] Joffre J., Hellman J., Ince C., Ait-Oufella H. (2020). Am. J. Respir. Crit. Care Med..

[cit6] Cochrane A., Albers H. J., Passier R., Mummery C. L., van den Berg A., Orlova V. V., van der Meer A. D. (2019). Adv. Drug Delivery Rev..

[cit7] Moses S. R., Adorno J. J., Palmer A. F., Song J. W. (2021). Am. J. Physiol..

[cit8] Sweeney M., Foldes G. (2018). Front. Cardiovasc. Med..

[cit9] Gaengel K., Genove G., Armulik A., Betsholtz C. (2009). Arterioscler., Thromb., Vasc. Biol..

[cit10] Aird W. C. (2007). Circ. Res..

[cit11] Muller W. A. (2003). Trends Immunol..

[cit12] Muller W. A. (2002). Lab. Invest..

[cit13] Aird W. C. (2012). Cold Spring Harbor Perspect. Med..

[cit14] Aird W. C. (2005). J. Thromb. Haemostasis.

[cit15] Pradhan S., Banda O. A., Farino C. J., Sperduto J. L., Keller K. A., Taitano R., Slater J. H. (2020). Adv. Healthcare Mater..

[cit16] Amaya R., Pierides A., Tarbell J. M. (2015). PLoS One.

[cit17] Roux E., Bougaran P., Dufourcq P., Couffinhal T. (2020). Front. Physiol..

[cit18] Baratchi S., Khoshmanesh K., Woodman O. L., Potocnik S., Peter K., McIntyre P. (2017). Trends Mol. Med..

[cit19] Qiu Y., Tarbell J. M. (2000). J. Vasc. Res..

[cit20] Arik Y. B., Buijsman W., Loessberg-Zahl J., Cuartas-Velez C., Veenstra C., Logtenberg S., Grobbink A. M., Bergveld P., Gagliardi G., den Hollander A. I., Bosschaart N., van den Berg A., Passier R., van der Meer A. D. (2021). Lab Chip.

[cit21] Lehoux S., Esposito B., Merval R., Tedgui A. (2005). Circulation.

[cit22] Namba M., Matsuo T., Shiraga F., Ohtsuki H. (2001). Ophthalmic Res..

[cit23] Haga M., Chen A., Gortler D., Dardik A., Sumpio B. E. (2003). Endothelium.

[cit24] Liu X. M., Ensenat D., Wang H., Schafer A. I., Durante W. (2003). FEBS Lett..

[cit25] Cummins P. M., von Offenberg Sweeney N., Killeen M. T., Birney Y. A., Redmond E. M., Cahill P. A. (2007). Am. J. Physiol..

[cit26] Hynes W. F., Pepona M., Robertson C., Alvarado J., Dubbin K., Triplett M., Adorno J. J., Randles A., Moya M. L. (2020). Sci. Adv..

[cit27] Kolesky D. B., Truby R. L., Gladman A. S., Busbee T. A., Homan K. A., Lewis J. A. (2014). Adv. Mater..

[cit28] Jimenez-Torres J. A., Peery S. L., Sung K. E., Beebe D. J. (2016). Adv. Healthcare Mater..

[cit29] Polacheck W. J., Kutys M. L., Tefft J. B., Chen C. S. (2019). Nat. Protoc..

[cit30] Adriani G., Ma D., Pavesi A., Kamm R. D., Goh E. L. (2017). Lab Chip.

[cit31] Vila Cuenca M., Cochrane A., van den Hil F. E., de Vries A. A. F., Lesnik Oberstein S. A. J., Mummery C. L., Orlova V. V. (2021). Stem Cell Rep..

[cit32] Herland A., van der Meer A. D., FitzGerald E. A., Park T. E., Sleeboom J. J., Ingber D. E. (2016). PLoS One.

[cit33] de Graaf M. N. S., Cochrane A., van den Hil F. E., Buijsman W., van der Meer A. D., van den Berg A., Mummery C. L., Orlova V. V. (2019). APL Bioeng..

[cit34] van Steen A. C. I., Kempers L., Schoppmeyer R., Blokker M., Beebe D. J., Nolte M. A., van Buul J. D. (2021). J. Cell Sci..

[cit35] de Graaf M. N. S. , PID-controller for microfluidic flow, https://github.com/mnsdegraaf/mfcb, (accessed 08-08-2022, 2022)

[cit36] Dessalles C. A., Ramon-Lozano C., Babataheri A., Barakat A. I. (2021). Biofabrication.

[cit37] Vollertsen A. R., Vivas A., van Meer B., van den Berg A., Odijk M., van der Meer A. D. (2021). Biomicrofluidics.

[cit38] Vollertsen A. R., de Boer D., Dekker S., Wesselink B. A. M., Haverkate R., Rho H. S., Boom R. J., Skolimowski M., Blom M., Passier R., van den Berg A., van der Meer A. D., Odijk M. (2020). Microsyst. Nanoeng..

[cit39] Vivas A., van den Berg A., Passier R., Odijk M., van der Meer A. D. (2022). Lab Chip.

[cit40] de Graaf M. N. S., Vivas A., van der Meer A. D., Mummery C. L., Orlova V. V. (2022). Micromachines.

[cit41] Chrobak K. M., Potter D. R., Tien J. (2006). Microvasc. Res..

[cit42] Oh K. W., Lee K., Ahn B., Furlani E. P. (2012). Lab Chip.

[cit43] Arik Y. B., de Sa Vivas A., Laarveld D., van Laar N., Gemser J., Visscher T., van den Berg A., Passier R., van der Meer A. D. (2021). ACS Biomater. Sci. Eng..

[cit44] Vivas A., IJspeert C., Pan J. Y., Vermeul K., den Berg A., Passier R., Keller S. S., van der Meer A. D. (2022). Adv. Mater. Technol..

[cit45] Yadav A. R., Sriram R., Carter J. A., Miller B. L. (2014). Mater. Sci. Eng., C.

[cit46] Roberts B., Haupt A., Tucker A., Grancharova T., Arakaki J., Fuqua M. A., Nelson A., Hookway C., Ludmann S. A., Mueller I. A., Yang R., Horwitz R., Rafelski S. M., Gunawardane R. N. (2017). Mol. Biol. Cell.

[cit47] Orlova V. V., van den Hil F. E., Petrus-Reurer S., Drabsch Y., Ten Dijke P., Mummery C. L. (2014). Nat. Protoc..

[cit48] Liberzon A. , Aubert D. L. M., Bachant P., Käufer T., Bauer J. A., Dallas B. V. C., Borg J. and Ranleu T., OpenPIV/openpiv-python: OpenPIV - Python (v0.22.2), (accessed 26-04-2022, 2022)

[cit49] Lawton P. F., Lee M. D., Saunter C. D., Girkin J. M., McCarron J. G., Wilson C. (2019). Front. Physiol..

[cit50] Tokuda S., Higashi T., Furuse M. (2014). PLoS One.

[cit51] Arganda-Carreras I., Fernández-González R., Muñoz-Barrutia A., Ortiz-De-Solorzano C. (2010). Microsc. Res. Tech..

[cit52] Sung J. H., Wang Y. I., Sriram N. N., Jackson M., Long C., Hickman J. J., Shuler M. L. (2019). Anal. Chem..

[cit53] Bischel L. L., Lee S. H., Beebe D. J. (2012). J. Lab. Autom..

[cit54] Kou S., Pan L., van Noort D., Meng G., Wu X., Sun H., Xu J., Lee I. (2011). Biochem. Biophys. Res. Commun..

[cit55] Sugiura S., Hattori K., Kanamori T. (2010). Anal. Chem..

[cit56] Cho Y. I., Cho D. J. (2011). Korean Circ. J..

[cit57] Poon C. (2022). J. Mech. Behav. Biomed. Mater..

[cit58] Shirinsky V. P., Antonov A. S., Birukov K. G., Sobolevsky A. V., Romanov Y. A., Kabaeva N. V., Antonova G. N., Smirnov V. N. (1989). J. Cell Biol..

[cit59] Zeinali S., Thompson E. K., Gerhardt H., Geiser T., Guenat O. T. (2021). APL Bioeng..

[cit60] Collins N. T., Cummins P. M., Colgan O. C., Ferguson G., Birney Y. A., Murphy R. P., Meade G., Cahill P. A. (2006). Arterioscler., Thromb., Vasc. Biol..

[cit61] Morel S., Schilling S., Diagbouga M. R., Delucchi M., Bochaton-Piallat M. L., Lemeille S., Hirsch S., Kwak B. R. (2021). Front. Physiol..

[cit62] Langille B. L. (2001). Microcirculation.

[cit63] van Geemen D., Smeets M. W., van Stalborch A. M., Woerdeman L. A., Daemen M. J., Hordijk P. L., Huveneers S. (2014). Arterioscler., Thromb., Vasc. Biol..

[cit64] Zhao S. M., Suciu A., Ziegler T., Moore J. E., Burki E., Meister J. J., Brunner H. R. (1995). Arterioscler., Thromb., Vasc. Biol..

[cit65] Vion A. C., Perovic T., Petit C., Hollfinger I., Bartels-Klein E., Frampton E., Gordon E., Claesson-Welsh L., Gerhardt H. (2020). Front. Physiol..

