# Supplementary material for: Multiplexed fluidic circuit board for controlled perfusion of 3D blood vessels-on-a-chip
Source: Lab Chip. 2022 Dec 9;23(1):168–81. doi: 10.1039/d2lc00686c (PMC9764810; doi:10.1039/d2lc00686c)
Supplement: LC-023-D2LC00686C-s001 [file LC-023-D2LC00686C-s001.pdf]

Electronic Supplementary Material (ESI) for Lab on a Chip.
This journal is © The Royal Society of Chemistry 2022

Electronic supplementary information

Multiplexed fluidic circuit board for controlled perfusion of 3D blood vessels-on-a-chip

Mees N. S. de Graaf[1], Aisen Vivas[2,3], Dhanesh G. Kasi[1,4,5], Francijna E. van den Hil[1], Albert van den Berg[3], Andries D. van der Meer[2], Christine L. Mummery[1,2], Valeria V. Orlova[1*]

[1] Department of Anatomy and Embryology, Leiden University Medical Center, 2333 ZA Leiden, The Netherlands

[2] Applied Stem Cell Technologies, 7500AE Enschede, University of Twente, The Netherlands

[3] BIOS Lab on a Chip Group, MESA+ Institute for Nanotechnology, Technical Medical Centre, Max Planck Institute for Complex Fluid Dynamics, University of Twente, 7500AE Enschede, The Netherlands

[4] Department of Human Genetics, Leiden University Medical Center, 2333 ZA Leiden, The Netherlands

[5] Department of Neurology, Leiden University Medical Center, 2333 ZA Leiden, The Netherlands

[*]Corresponding author e-mail address: v.orlova@lumc.nl

Supplementary Figures

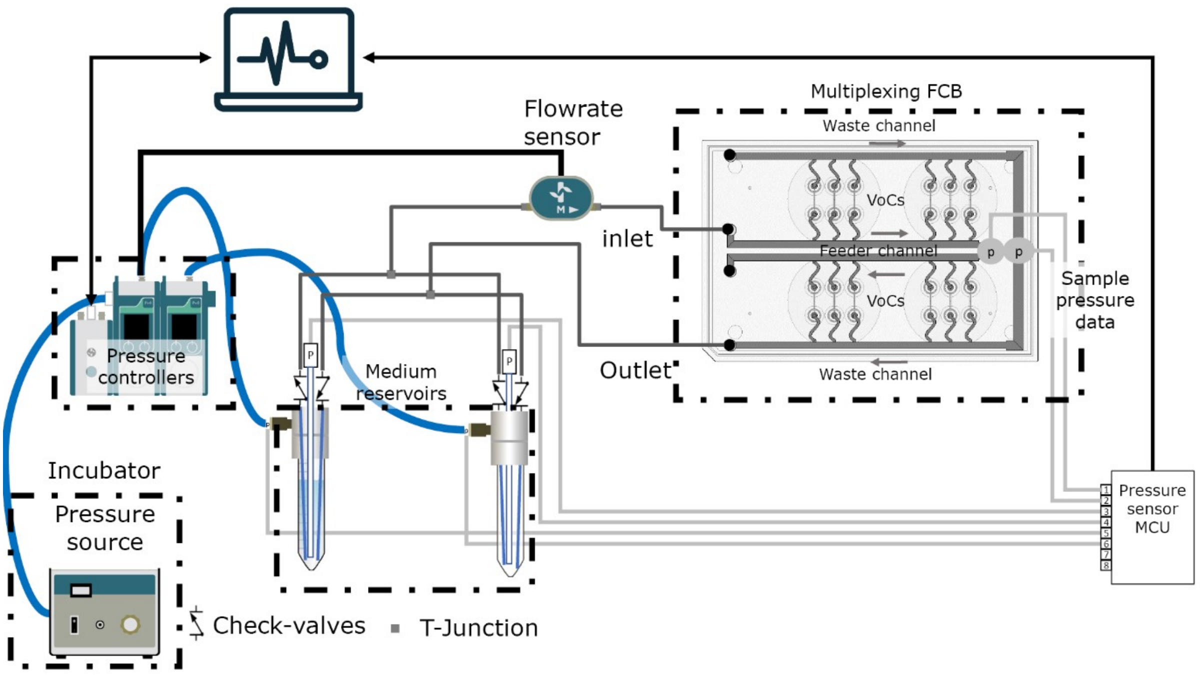

Fig. s1 Fluidic circuit used for long-term perfusion. It requires the addition of two T or Y-connectors and results in a unidirectional flow.

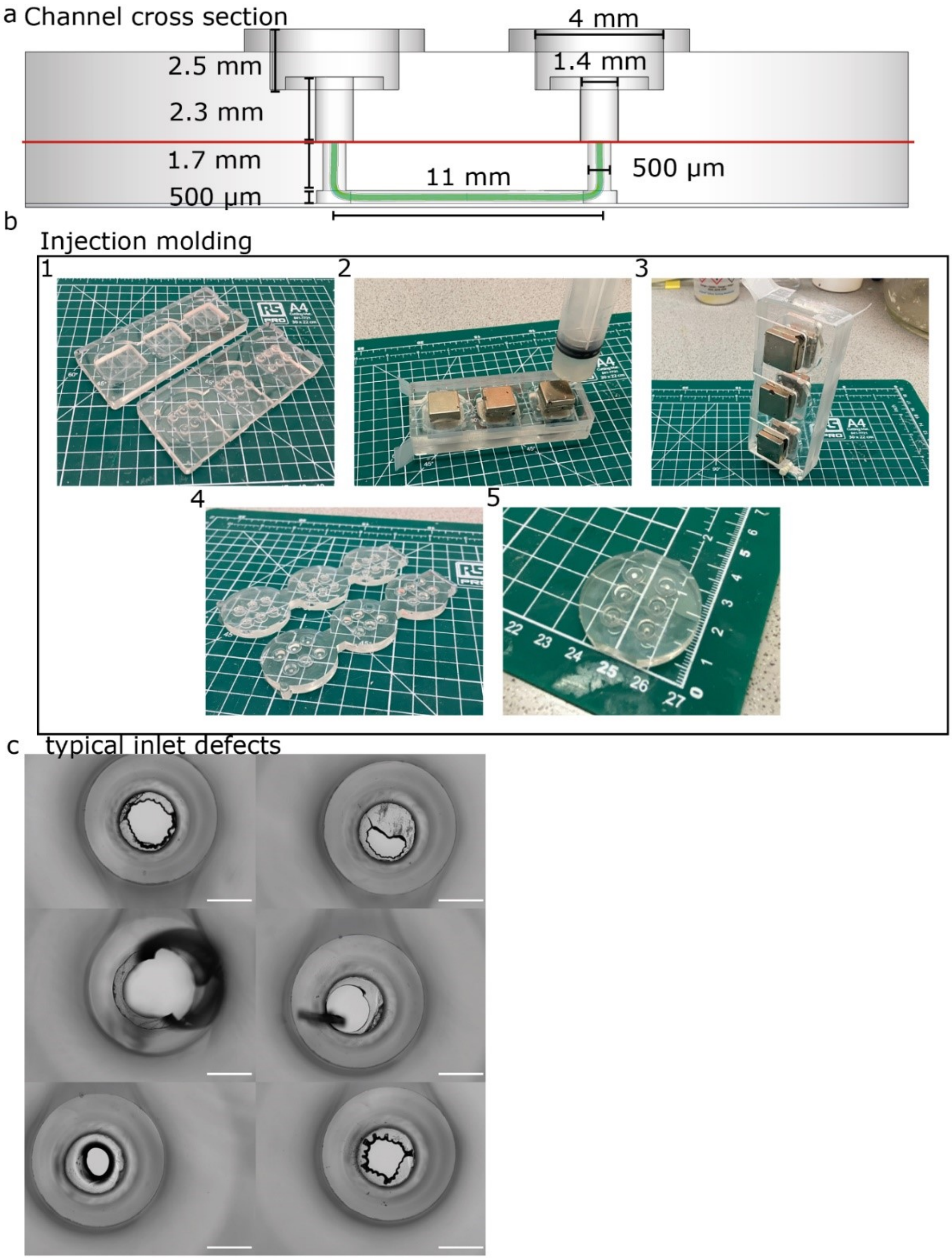

Fig. s2 Chip design and Microfabrication using injection moulding. (a) Chip design and channel dimensions, green represents the modelled lumen, the red line indicates where the two mould halves have contact. (b) Injection moulding. Two separate mould halves show the fluidic channels and medium reservoirs (1). Injection moulds are assembled using strong magnets and PDMS is injected using a syringe (2). The injection mould is placed vertically and allowed to set at room temperature for at least 16 hours (3) followed by 1 hour at 75 °C. Magnets are removed and the PDMS is peeled of (4) excess PDMS is cut off (5) and the chip is assembled with air plasma treatment. (c) Defects

at the contact points of the injection-moulds result in small membranes of various sizes.

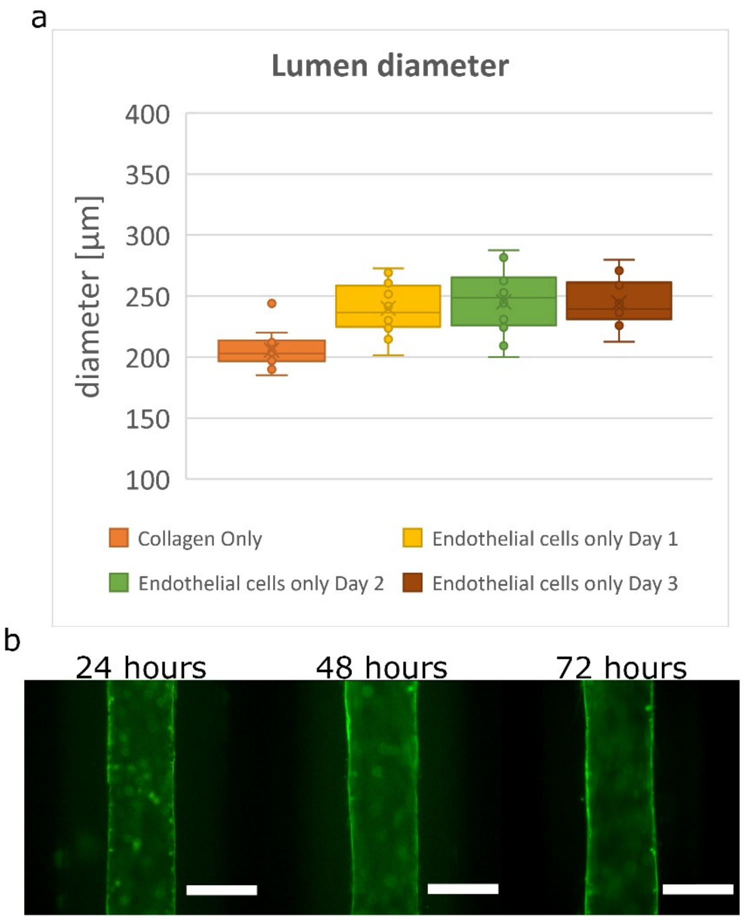

Fig. s3 (a)Boxplot of VFP luminal diameter. Typical luminal diameter ranges of the 3D-VoC in a single experiment shows a diameter expansion after cell seeding. The seeded lumens remain constant in diameter for at least 3 days.(b) representative images of lumen at day 1, day 2 and day 3. Scalebar 200 µm

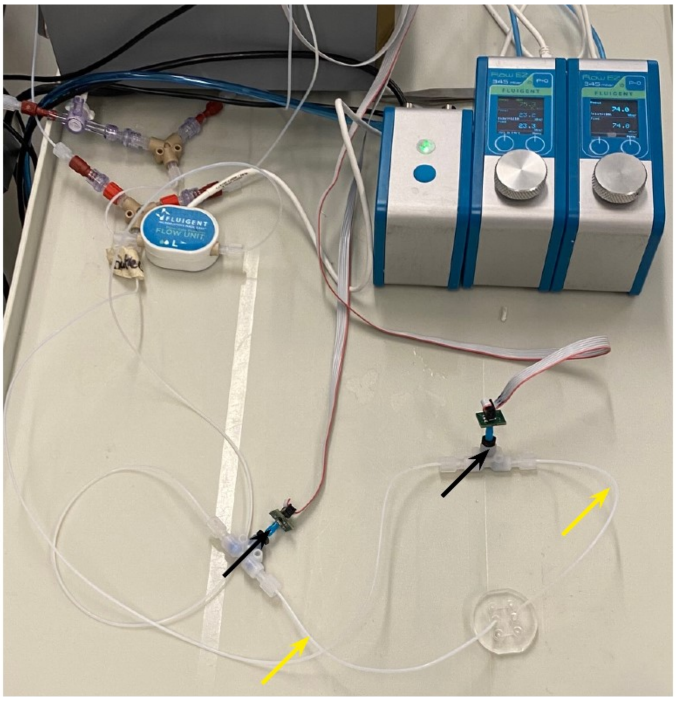

Fig. s4 Set-up for measuring individual shear stress. A total of 52 cm of tubing (yellow arrows) was used and placed between the pressure sensors (black arrows) and the samples. The pressure difference was controlled by custom software.

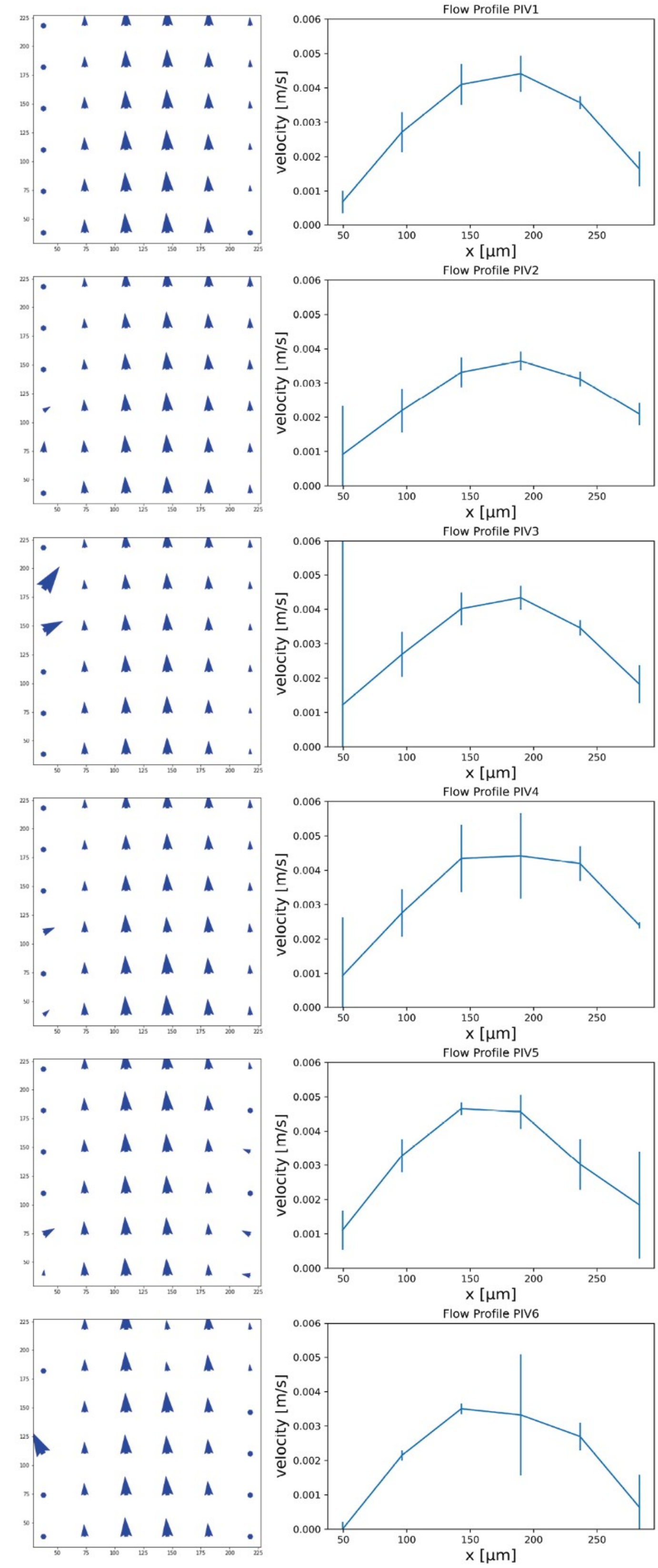

Fig. s5 PIV Vector fields of all analysed samples.

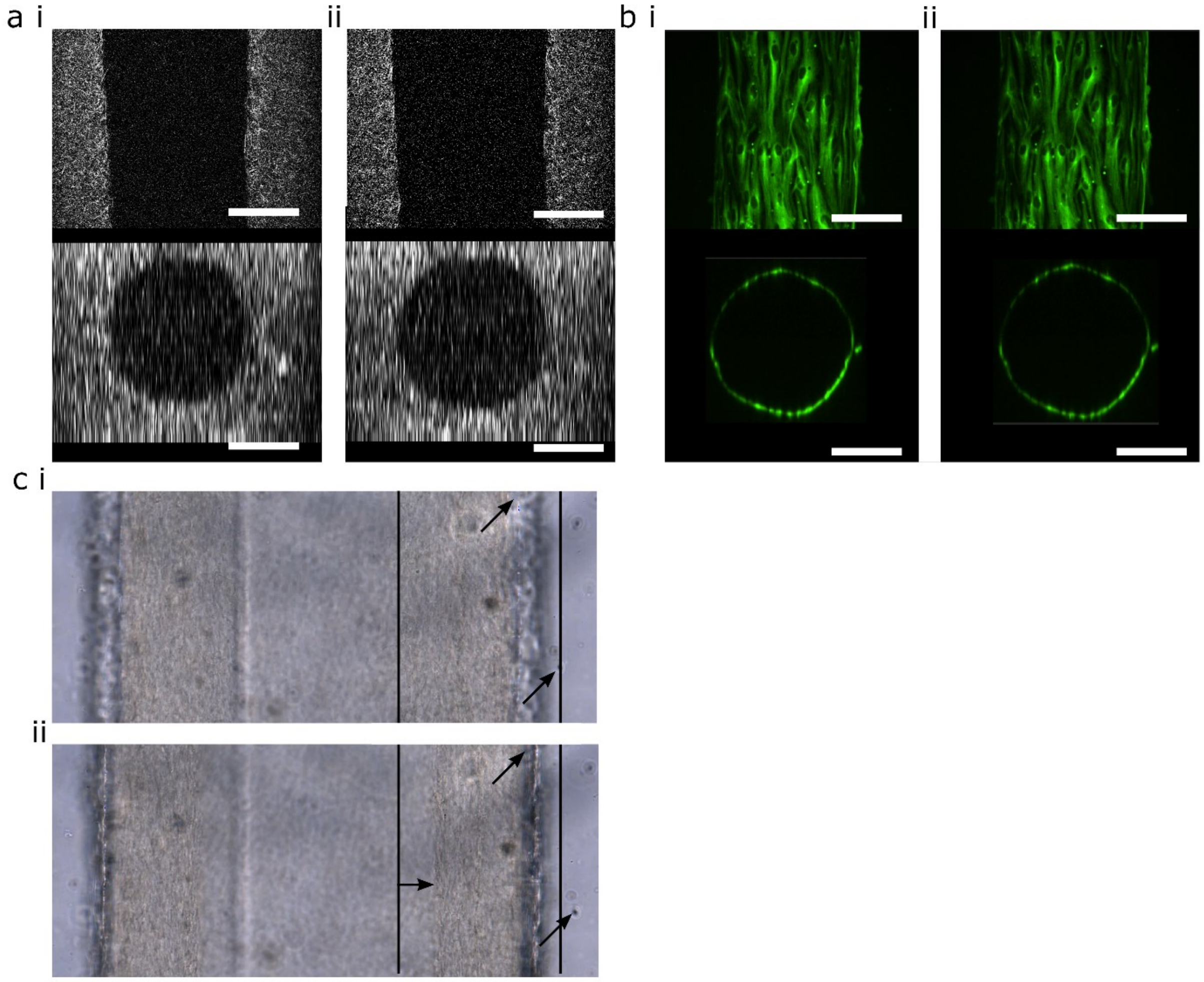

Fig. s6 Imaging vascular compliance in 3D. (a) 2p-SHG image of the middle frame and a cross section of the lumen at P = 0 mbar (i) and P = 345 mbar (ii), see video 3 for the animated sequence. (b) Spinning disc confocal reconstruction bottom half of the lumen cross section of the lumen at P = 0 mbar (i) and P = 345 mbar (ii), see video 4 for the animated sequence. (c) Frames of video 2 to highlight deformation of the lumen and the PDMS showing P = 0 mbar (i) and P = ±1000 mbar (ii). Arrows and lines are visual references to aid comparison. Scale bars 100 μm.

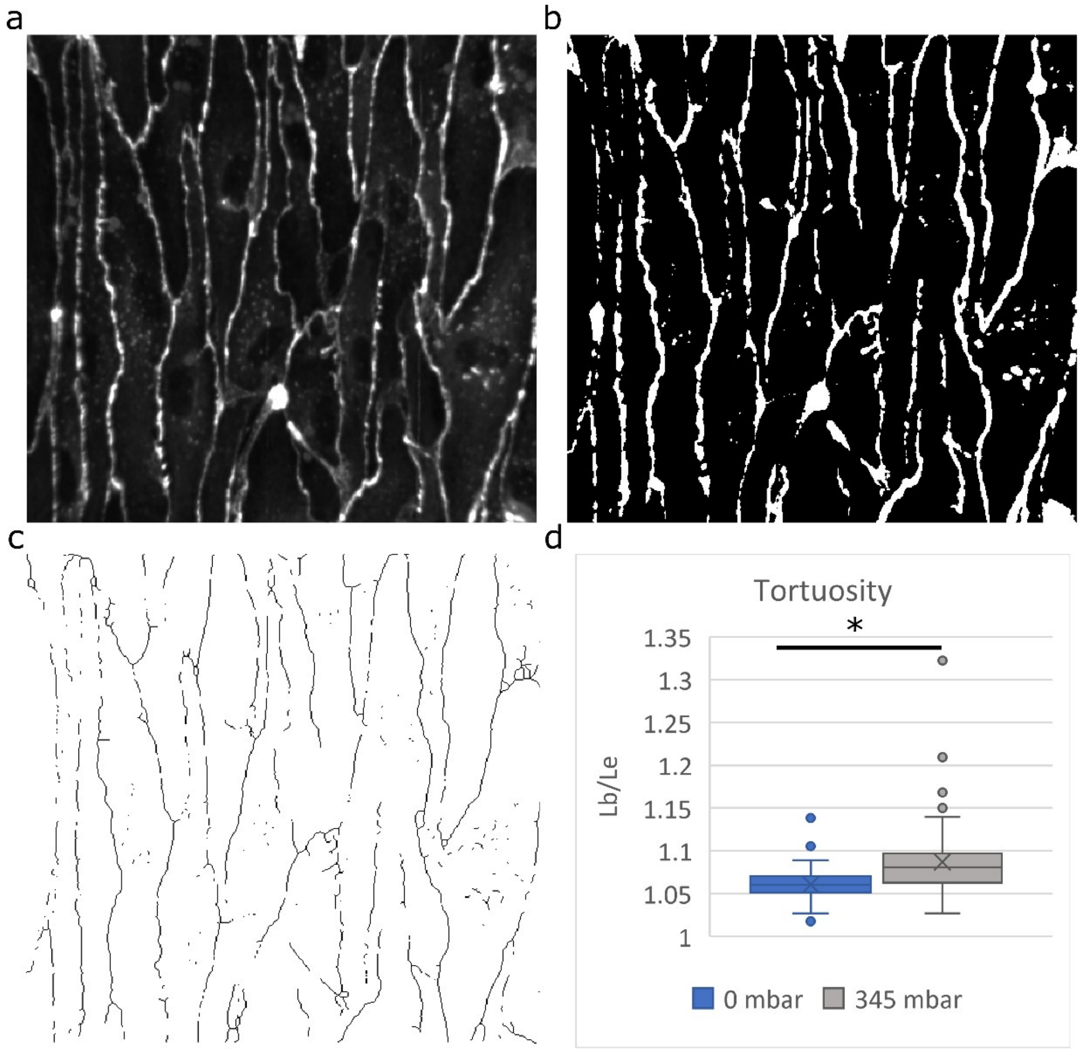

Fig. s7 Quantification of the tortuosity index of adherens junctions (a) Cell junctions visualized using VE-cadherin marker (b) Threshold image of VE-cadherin (c) Skeletonized image of VE-cadherin (d) tortuosity index is calculated by dividing the length of branch (Lb) by the Euclidean distance of that branch(Le). Quantification based on one sequence, one point is one cell junction* indicates P<0.001

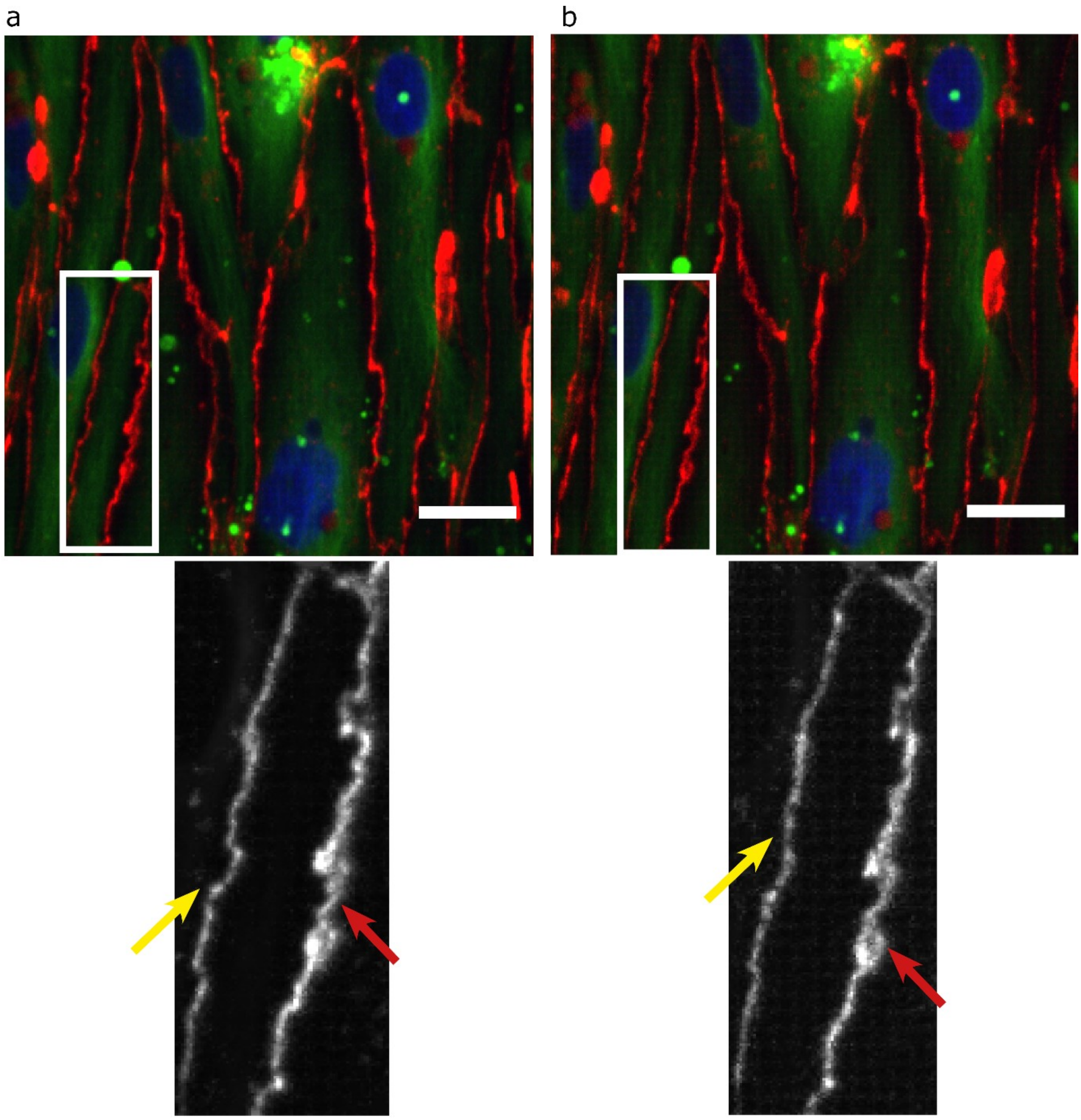

Fig. s8 Confocal reconstruction of live TUBA1B-eGFP-ECs (green) co-stained for adherens junctional marker (VE-cadherin, in red) and nuclei (Hoechst, in blue) at high pressure (345 mbar) (a) and at low pressure (0 mbar) (b). Inserts give example of morphological changes in VE-cadherin cell junctions at high and low pressure. Borders go back to a straight line (yellow arrows) dislocated junctions remain jagged (red arrows). Scale bar 20μm.

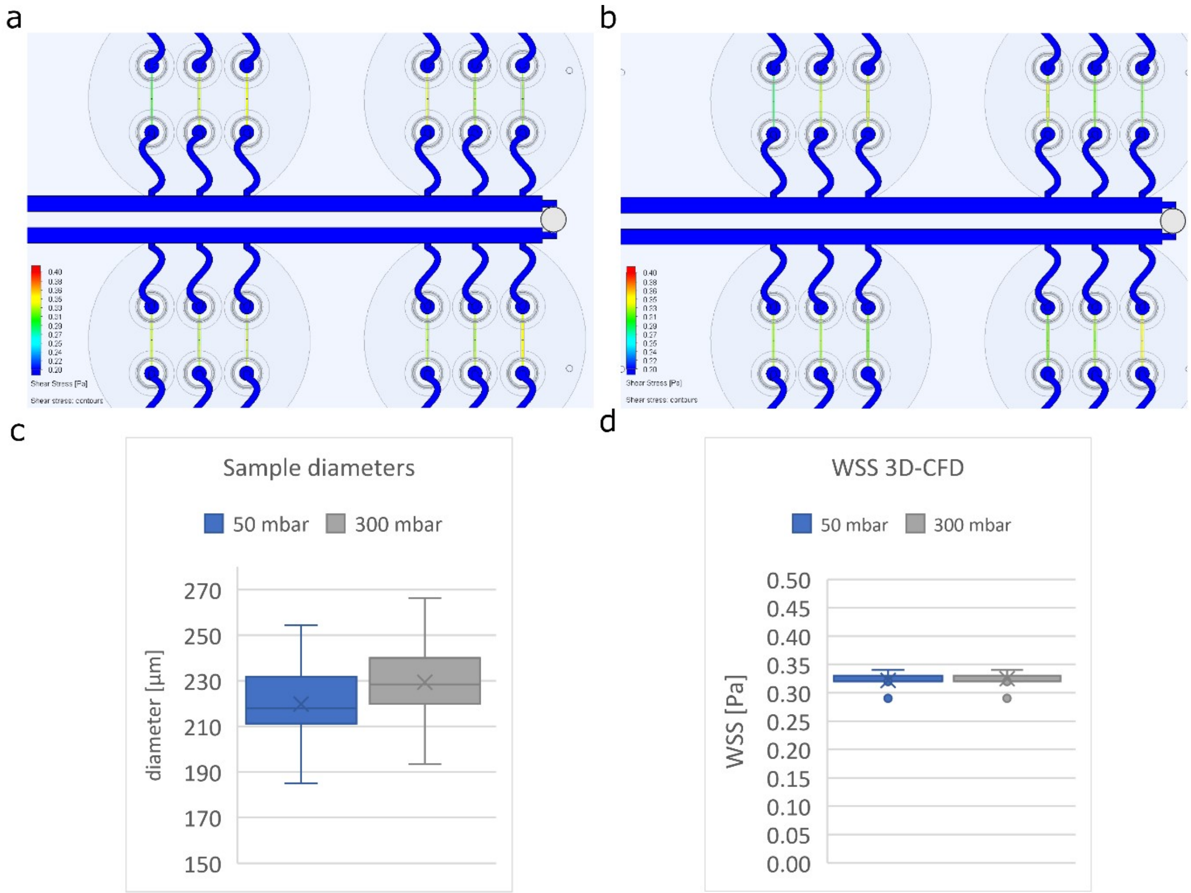

Fig. s9 CFD model of perfusion experiment (a) with an internal pressure of 50 mbar of internal pressure (b) with 300 mbar of internal pressure (c) Boxplot of all sample diameters at specified internal pressure (d) Boxplot of resulting WSS at specified internal pressure.

Supplementary Videos

Video 1 Animation of 3D-CFD model showing the velocity stream lines.

Video 2 Brightfield scaffold only. Pressure was manually applied using a syringe up to 1 bar.

Video 3 2p-SHG sequence of scaffold only of pressure ramp. Frame 1, P = 0 mbar; frame 2, P = 345 mbar.

Video 4 3D-Confocal sequence of the TUB1a-mGFP-hiPSC-ECs pressure ramp. Frame 1, P = 0 mbar; frame 2, P = 345 mbar.

Video 5 Brightfield pressure ramp of the TUB1a-mGFP-hiPSC-ECs monolayer. P = 0-345 mbar, 25 mbar pressure increment per frame, last frame returns to P = 0 mbar.

Video 6 Widefield fluorescent signal of the TUB1a-mGFP-hiPSC-ECs monolayer analysed using the VasoTracker software (blue lines).

Video 7 Confocal reconstruction of pressure ramp. P = 0-50-75-100 mbar. Green: TUB1a-mGFP-hiPSC-ECs, red: VE-Cadherin, blue: Hoechst. Histograms of the red channel were equalized per frame for the reconstruction. Note: last frame shows that the VE-cadherin signal is completely lost due to photobleaching of the fluorophore.

Video 8 Confocal reconstruction of pressure ramp. Frame 1, P = 0 mbar; frame 2, P = 150 mbar; frame 3, P = 345 mbar, frame 4, P = 325 mbar with 1 Pa of WSS. Green: TUB1a-mGFP-hiPSC-ECs, red: VE-Cadherin, blue: Hoechst.

Deriving the equation of optimum resistance of the branch channels to minimize wall shear stress

Hagen–Poiseuille's law for pressure driven flow:

$$\Delta P = R_h Q \qquad \text{Eq. s1}$$

Pressure difference for a serial connected resistance:

$$\Delta P = Q\ (R_h^{vessel} + R_h^{\tau EQ}) \qquad \text{Eq. s2}$$

WSS for circular lumen with given diameter and flowrate

$$\tau\ = \frac{32\ \mu\ Q}{\pi\ d^3} \qquad \text{Eq. s3}$$

Rewrite to flowrate for given diameter and WSS

$$Q\ = \frac{\tau\ \pi\ d^3}{32\ \mu} \qquad \text{Eq. s4}$$

Combine Eq. s4 in Eq. s2 with R_h for a circular 3D-VoC assuming uniform diameter of the sample, negligible interstitial flow

$$\Delta P = (\frac{\tau\ \pi\ d^3}{32\ \mu})(128\ \mu \frac{l_{vessel}}{\pi d^4} + R_h^{\tau EQ}) \qquad \text{Eq. s5}$$

Set WSS d_{min} equal to WSS d_{max} given equal dP (Eq. s6)

$$\left(\frac{\tau\ \pi\ d_{min}^{\ 3}}{32\ \mu}\right)\left(128\ \mu \frac{l_{min}}{\pi d\ [illegible]^{\ 4}} + R_h^{\tau EQ}\right) = \left(\frac{\tau\ \pi\ d_{max}^{\ 3}}{32\ \mu}\right)\left(128\ \mu \frac{l_{max}}{\pi d\ [illegible]^{\ 4}} + R_h^{\tau EQ}\right)$$

Eq. s6 can be reduced to Eq. s7

$$\frac{4\ l_{min}}{d_{min}} + \left(\frac{\pi\ d_{min}^{\ 3}}{32\ \mu}\right) R_h^{\tau EQ} = \frac{4\ l_{max}}{d_{max}} + \left(\frac{\pi\ d_{max}^{\ 3}}{32\ \mu}\right) R_h^{\tau EQ}$$

Optimal τEQ resistance can then be expressed in terms of sample diameters

$$R_h^{\tau EQ} = \frac{128\ \mu(\frac{l_{min}}{d_{min}} - \frac{l_{max}}{d_{max}})}{[illegible]} \qquad \text{Eq. s8}$$

τEQ resistor dimensions of a rectangular channel:

$$\frac{l_{ressistor}}{\text{[illegible]}} = \frac{32(\frac{l_{min}}{d_{min}} - \frac{l_{max}}{d_{max}})}{3\pi(d_{\text{[illegible]}}^{3} - d_{\text{[illegible]}}^{3})} \quad \text{Eq.s9}$$

τEQ resistor dimensions of a circular tube:

$$\frac{l_{res}}{\text{[illegible]}} = \frac{(\frac{l_{min}}{d_{min}} - \frac{l_{max}}{d_{max}})}{\text{[illegible]}} \quad \text{Eq.s10}$$

Deriving equation: Required ΔP for given WSS

Serie circuit of sample and τEQ resistor combined with Eq. s4

$$\Delta P = (\frac{\tau\pi d^3}{32\mu})(128\,\mu\frac{L_{vessel}}{\pi d^4} + 12\mu\frac{L}{\text{[illegible]}}) \quad \text{Eq. s5}$$

$$\Delta P = (\frac{\tau 4 L_{vessel}}{d} + \frac{\tau\, 12\,\pi d^3 L}{\text{[illegible]}})$$

$$\Delta P = \tau(4\frac{L_v}{d} + \frac{3\,\pi\,d^3 L_{res}}{8\,w\,h^3(1 - 0.63\frac{h}{w})}) \quad \text{Eq. 1}$$

Equations wall shear stress used for Fig. 2c

Fixed Q red plot:

$$\tau = \frac{32\,\mu\,Q}{\pi\,d^3} \quad \text{Eq.s3}$$

τ: WSS

Q: flowrate = 90 $\frac{\mu l}{min}$

μ: viscosity= 0.79 Pa s

d: diameter range= 180-300 μm

Fixed P (green plot):

$$\tau = \frac{\Delta P\, d}{4\, l_{vessel}} \qquad \text{Eq. s11}$$

ΔP: Fixed pressure difference= 237 [Pa]
d: Diameter range= 180-300 μm
l:length =1.43 cm

τEQ + Fixed ΔP blue plot:

$$\tau = \frac{\Delta P}{\left(4\frac{l_{vessel}}{d} + \frac{3\pi d^3 L_{res}}{8wh^3\left(1 - 0.63\frac{h}{w}\right)}\right)} \qquad \text{Eq. s12}$$

Fixed ΔP: 333 [Pa]
d:diameter range= 180-300 μm
l=1.43 cm
L_{res},w,h= dimensions of resistor listed in table s1 [m]

Table s1 channel dimensions FCB

			Viscosity [Pa s]	7.90E-04
FCB dimensions	Length [m]	Width [m]	Height [m]	R_h [Pa s m^{-3}]
Feeder channel	0.2	0.0025	0.002	1.90E+08
Feeder loop	0.01	0.0012	0.002	4.40E+07
			Total	2.34E+08
Waste channel	0.2	0.0025	0.002	1.90E+08
Waste loop	0.082	0.0035	0.002	4.33E+07
			Total	2.34E+08
τEQ-Resistors	0.0171	0.001	0.0002	2.32E+10
3D-VoC	length [m]	d [μm]		R_h including chip resistance
Small	0.0143	180		4.38E+11
Medium	0.0143	240		1.39E+11
Large	0.0143	300		5.68E+10

Table s2 Dimensions lumen expansion

	Scaffold-only		EC-monolayer	
Pressure [mbar]	p0	p345	p0	p345
Lumen width [µm]	200	220	196	206
Lumen height [µm]	195	215	197	208
Ratio (w/h)	1.03	1.02	0.99	0.99
